# Cardiac magnetic resonance -detected myocardial injury is not associated with long-term symptoms in patients hospitalized due to COVID-19

**DOI:** 10.1371/journal.pone.0282394

**Published:** 2023-03-08

**Authors:** Aria Yar, Valtteri Uusitalo, Satu M. Vaara, Miia Holmström, Aino-Maija Vuorinen, Tiina Heliö, Riitta Paakkanen, Sari Kivistö, Suvi Syväranta, Johanna Hästbacka

**Affiliations:** 1 Radiology, HUS Diagnostic Center, Helsinki University Hospital and University of Helsinki, Helsinki, Finland; 2 Clinical Physiology and Nuclear Medicine, HUS Diagnostic Center, Helsinki University Hospital and University of Helsinki, Helsinki, Finland; 3 Heart and Lung Center, Helsinki University Hospital and University of Helsinki, Helsinki, Finland; 4 Department of Anesthesiology, Intensive Care and Pain Medicine, Helsinki University Hospital, and University of Helsinki, Helsinki, Finland; King’s College London, UNITED KINGDOM

## Abstract

**Background:**

Long-term symptoms are frequent after coronavirus disease 2019 (COVID-19). We studied the prevalence of post-acute myocardial scar on cardiac magnetic resonance imaging (CMR) in patients hospitalized due to COVID-19 and its association with long-term symptoms.

**Materials and methods:**

In this prospective observational single-center study, 95 formerly hospitalized COVID-19 patients underwent CMR imaging at the median of 9 months after acute COVID-19. In addition, 43 control subjects were imaged. Myocardial scar characteristic of myocardial infarction or myocarditis were noted from late gadolinium enhancement images (LGE). Patient symptoms were screened using a questionnaire. Data are presented as mean ± standard deviation or median (interquartile range).

**Results:**

The presence of any LGE was higher in COVID-19 patients (66% vs. 37%, p<0.01) as was the presence of LGE suggestive of previous myocarditis (29% vs. 9%, p = 0.01). The prevalence of ischemic scar was comparable (8% vs. 2%, p = 0.13). Only two COVID-19 patients (7%) had myocarditis scar combined with left ventricular dysfunction (EF <50%). Myocardial edema was not detected in any participant. The need for intensive care unit (ICU) treatment during initial hospitalization was comparable in patients with and without myocarditis scar (47% vs. 67%, p = 0.44). Dyspnea, chest pain, and arrhythmias were prevalent in COVID-19 patients at follow-up (64%, 31%, and 41%, respectively) but not associated with myocarditis scar on CMR.

**Conclusions:**

Myocardial scar suggestive of possible previous myocarditis was detected in almost one-third of hospital-treated COVID-19 patients. It was not associated with the need for ICU treatment, greater symptomatic burden, or ventricular dysfunction at 9 months follow-up. Thus, post-acute myocarditis scar on COVID-19 patients seems to be a subclinical imaging finding and does not commonly require further clinical evaluation.

## Introduction

Myocardial injury is common among patients hospitalized due to coronavirus disease 2019 (COVID-19) [1, 2]. The causes are multifactorial, including systemic inflammation, endothelitis, myocardial ischemia, heart failure, Takotsubo cardiomyopathy, exacerbation of chronic cardiac diseases, and rarely, myocarditis [3, 4]. In recent studies, long-term symptoms potentially caused by cardiovascular disease have been prevalent in patients after COVID-19 [5–7]. Furthermore, the risk of various cardiovascular conditions remains elevated for at least a year after the initial infection [6].

Cardiac magnetic resonance (CMR) imaging is the gold standard of the non-invasive detection of myocardial diseases due to its ability to detect both myocardial inflammation and scar [8, 9] accurately. Interestingly, both ischemic and non-ischemic myocardial scars detected as late-gadolinium enhancement (LGE) have been frequent in COVID-19 patients [10]. However, the association of myocardial scar with long-term symptoms after COVID-19 is currently unclear.

In this prospective observational study, we evaluated the prevalence of myocardial scar, myocardial inflammation, and cardiac dysfunction in patients hospitalized due to COVID-19 after nine months of follow-up. We screened study participants for cardiovascular symptoms to evaluate the clinical significance of non-ischemic left ventricular scar suggestive of previous myocarditis in particular. The study’s primary aim was to investigate the prevalence of CMR abnormalities in COVID-19 patients and whether they are associated with a long-term symptomatic burden at long-term follow-up in COVID-19 patients.

## Materials and methods

This is a prospective observational study conducted between November 2020 and September 2021 in the Helsinki University Hospital. Patients hospitalized for a confirmed (polymerase chain reaction or SARS-CoV-2 antibody test) COVID-19 between March and December 2020 were recruited six months after hospital discharge. Eligible intensive care survivors and patients from a pulmonology ward and follow-up outpatient clinic were invited to participate in the study. A control group with no history of COVID-19 was recruited by media announcements. Our inclusion criteria were Finnish as the language of business and age ≥18. Exclusion criteria for the study were pregnancy, severe renal failure and previous allergic reaction to the gadolinium contrast, severe claustrophobia, and ferromagnetic implants affecting CMR imaging. Due to the concomitant participation in a neuropsychological substudy, previous major neurological comorbidity, neurodegenerative disease, developmental disability, and impaired hearing and vision were also exclusion criteria to the study. Clinical and laboratory variables at the time of hospitalization were collected from the electronic health records. At the time of CMR imaging, patients were screened for cardiovascular symptoms using a questionnaire, and they underwent laboratory testing, including high-sensitivity troponin T (hs-TnT), N-terminal pro B*-*type natriuretic peptide (NT-ProBNP), C-reactive protein (CRP) and serum creatinine. All study participants gave written informed consent for participating the study. The study protocol was approved by the Helsinki University Hospital’s ethics board of the Ethics board of Helsinki University Hospital (decision number HUS-1949-2020), and institutional permission was granted by the Department of Anesthesiology, Intensive Care and Pain Medicine (decision number HUS/200/2020). The study protocol is registered at clinicaltrials.gov (NCT04864938). The patients and control subjects were treated according to the principles of the Declaration of Helsinki.

### Cardiac magnetic resonance imaging

The imaging studies were conducted using a standard 1.5T CMR-scanner (MAGNETOM AvantoFit, Siemens Healthineers, Erlangen, Germany) according to international guidelines [11]. LGE imaging was performed 10 minutes after an intravenous injection of 0.15 mmol/kg of gadoterate meglumine (Dotarem®) using a phase-sensitive inversion‐recovery technique.

Ventricular volumes and left ventricular mass were traced from short-axis cine images with papillary muscles included in the blood pool. The Left and right atrial areas were traced on the 4-chamber image. Cardiac chamber volumes were normalized to the body-surface area. Myocardial edema was assessed using short Tau inversion recovery (STIR) imaging and T2-mapping [11, 12]. The presence of myocardial scar was evaluated from LGE images and divided into ischemic and non-ischemic based on the standard accepted clinical criteria by a European Association of Cardiovascular Imaging (EACVI) level 3 -certified cardiac radiologist (MH) [12]. In patients with non-ischemic left ventricular LGE, previous myocarditis was suspected when the scar was unexplained by physiological, or risk factor-related fibrosis and characteristic of myocarditis [9]. The clinical significance of abnormal CMR findings was assessed by a cardiologist (TH and RP) and patients were subsequently referred to a cardiology outpatient clinic, as necessary. Medis Suite Qmass 8.1 software was used for analyses (Medis Medical Imaging Systems B.V., Leiden, The Netherlands).

### Statistical analyses

Categorical variables are reported as numbers and percentages. Continuous variables are reported as mean ± standard deviation and median (interquartile range) for normally distributed and skewed data. The statistical comparisons were made in COVID-19 patients with and without different subtypes of LGE and in patients with and without COVID-19 hospitalization. The sample size of the study was assessed post-hoc by the method described by Fleiss et al [13]. The study aimed at recruiting 100 COVID-19 patients and 50 control subjects. The prevalence of scar characteristic for myocarditis was 29% in COVID-19 patients and 9% in control subjects. The recruitment ratio of control subjects to COVID-19 patients was 0.5. Required sample size to detect a difference in myocarditis scar prevalence with 80% power and a type I statistical error rate of 0.05 is 140 patients, of which 93 would be COVID-19 patients and 47 control subjects (95 and 43 in the final study population). The rare incidences of missing data were considered random and not significant for the study results. CMR analyses and the symptom questionnaire were unblinded for patients’ previous medical conditions. Student’s T-test was used to compare variables with normal distribution and the Mann-Whitney U test for variables with non-normal distribution, including patient characteristics, laboratory variables, and imaging findings in COVID-19 patients and control subjects. Categorical variables were compared using the Chi-squared test of independence. Two-tailed p-values of <0.05 were considered statistically significant. The analyses were performed using SPSS statistics 27 (SPSS Inc, Chicago, IL).

## Results

### Patient characteristics

**Fig 1:**
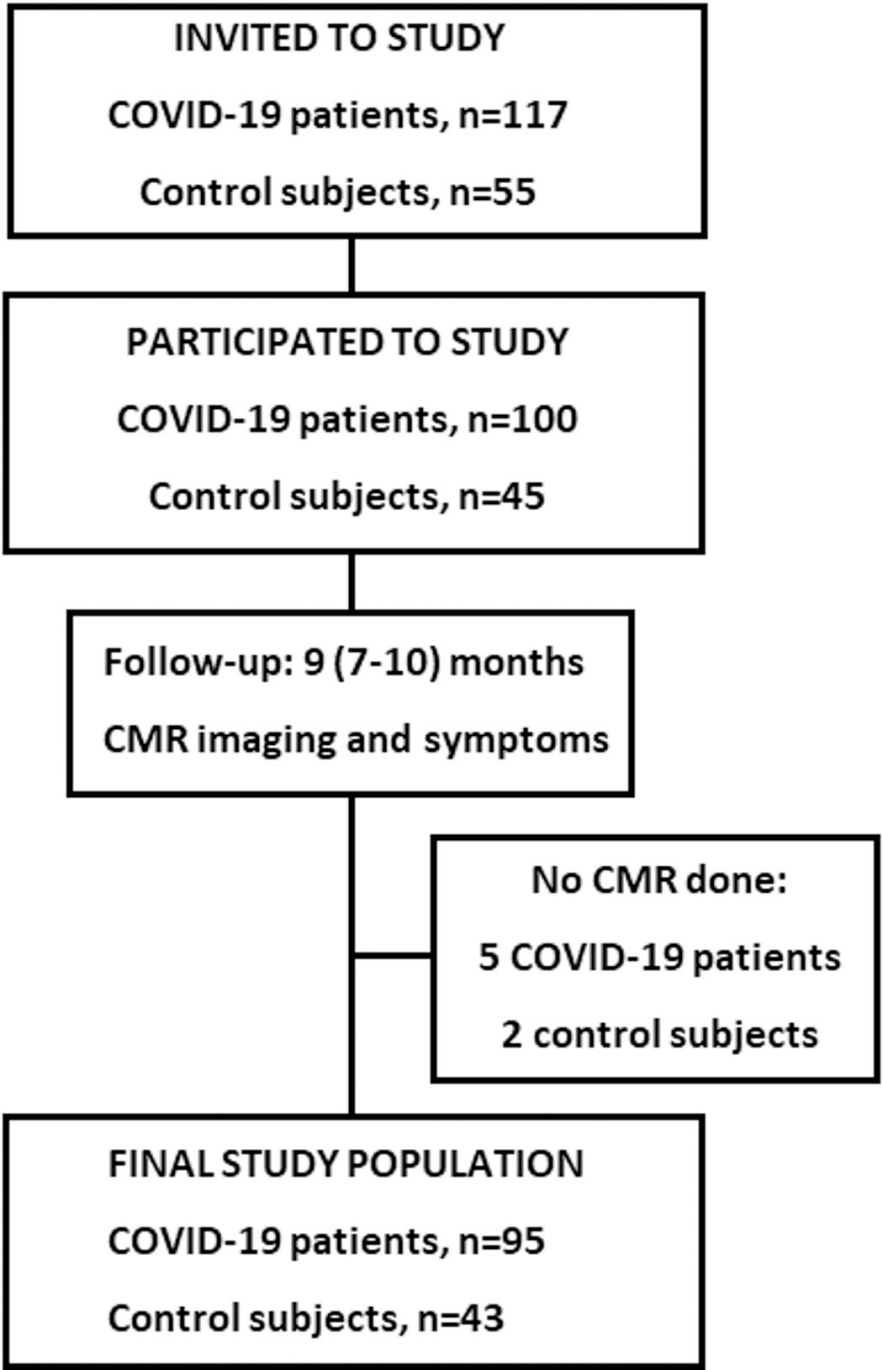
A flowchart on the patient enrollment to the study. CMR = cardiac magnetic resonance; COVID-19 = coronavirus disease 2019.

The study population consisted of 95 patients hospitalized due to COVID-19 and 43 control subjects (Fig 1). A total of 56 individuals (59%) received intensive care during the acute phase of COVID-19. The clinical characteristics of patients and control subjects are summarized in Table 1.

**Table 1:** Clinical characteristics of COVID-19 patients and control group during the initial hospitalization.

Characteristic	N	COVID-19 (n = 95)	Control (n = 43)	P-value
*Demographics*				
Female sex	138	49 (52%)	19 (44%)	0.42
Age (y)	138	59 ± 10	55 ± 13	0.052
Body mass index (kg/m^2^)	138	29.3 ± 4.6	26.4 ± 3.5	<0.001
Smoking	137	5 (5%)	5 (12%)	0.17
*Associated diseases*				
Hypertension	138	45 (47%)	9 (21%)	<0.01
Hypercholesterolemia	137	41 (44%)	17 (40%)	0.65
Diabetes	138	11 (12%)	0 (0%)	0.02
Coronary artery disease	137	9 (9%)	0 (0%)	0.04
Atrial fibrillation	137	7 (7%)	0 (0%)	0.09
Asthma	138	15 (16%)	3 (7%)	0.16
*Selected laboratory values*				
Elevated troponin	73	73 (22%)	-	
C-reactive protein (mg/l)	88	173 (108–236)	-	
Glomerular filtration rate (ml/min) *	138	90 (81–98)	92 (83–104)	0.17
*COVID-19-targeted treatment*				
Hospitalization	138	95 (100%)	0 (0%)	
ICU treatment	138	56 (59%)	0 (0%)	
Corticosteroid	94	14 (15%)	-	
Anticoagulation	94	90 (96%)	-	
COVID-19 vaccination**	120	17 (21%)	21 (55%)	<0.001

Data are presented as number of patients (%), median (interquartile range), or mean ± SD.

*At the time of CMR imaging.

**Any vaccination received before follow-up. COVID-19 = coronavirus disease 2019; ICU = intensive care unit.

### Results of cardiac imaging

**Fig 2:**
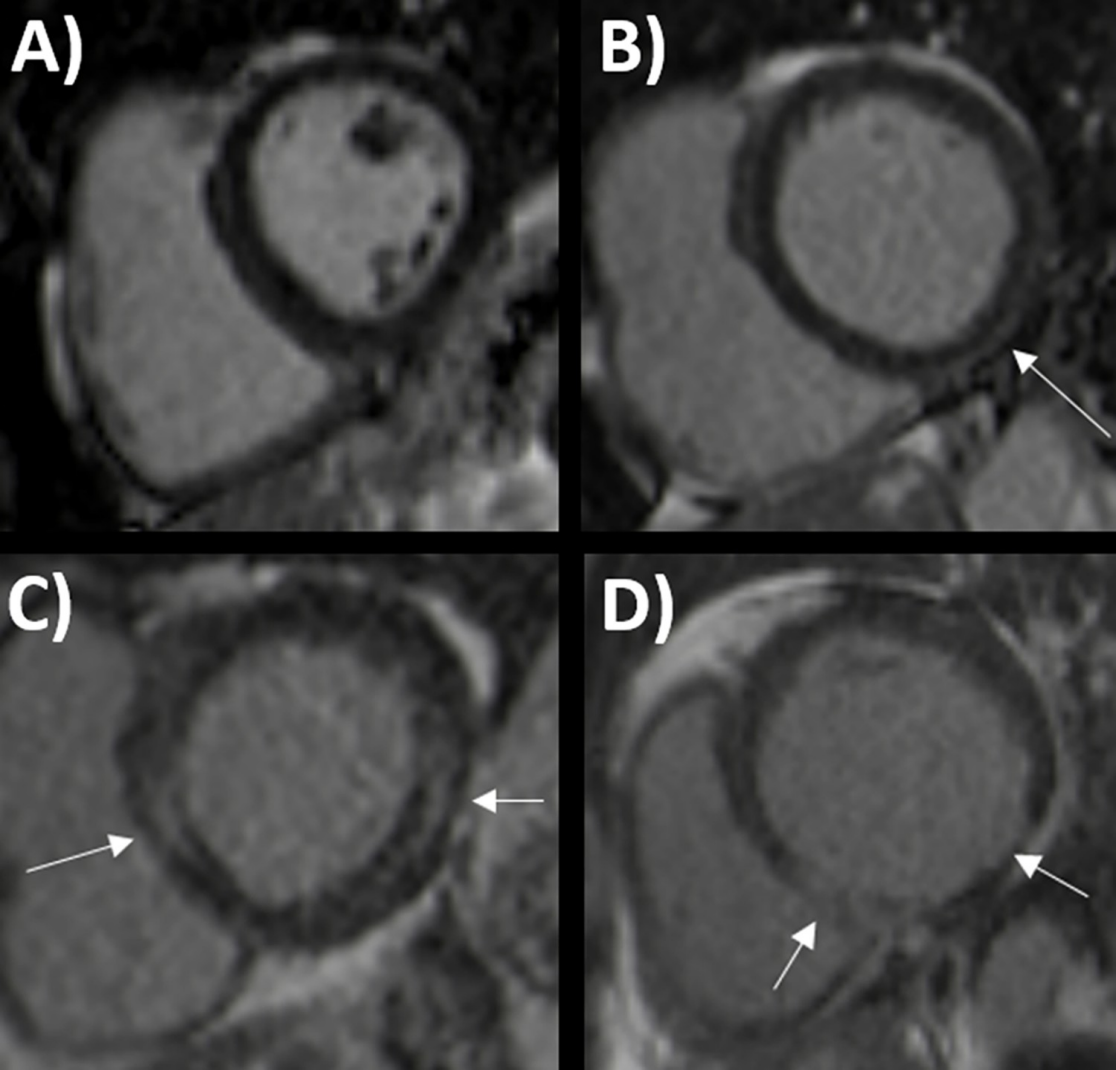
Examples of cardiac magnetic resonance imaging findings in patients with coronavirus disease 2019 (COVID-19). Normal left ventricle (LV) without late gadolinium enhancement (LGE) (**A**). Subepicardial LGE in inferolateral LV wall suggestive of previous myocarditis (**B**). More severe LGE with a septal and inferolateral non-ischemic pattern concordant with previous myocarditis (**C**). The inferior wall has extensive subendocardial and transmural scarring due to previous myocardial infarction (**D**). White arrow = LGE.

**Fig 3:**
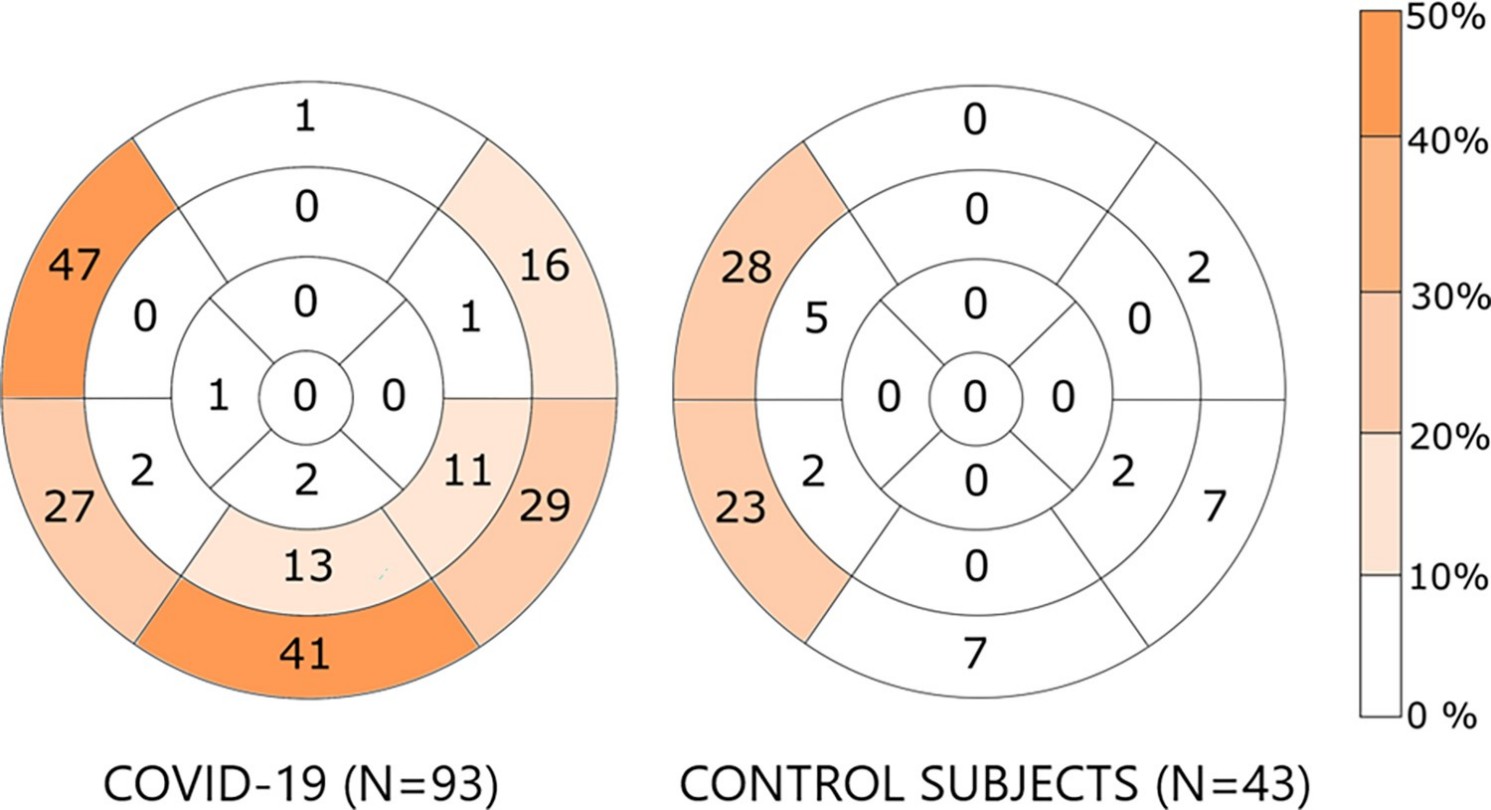
Localization of myocardial late gadolinium enhancement on magnetic resonance imaging in patients with and without coronavirus disease 2019 hospitalization. Localization of late gadolinium enhancement on magnetic resonance images in individuals with and without coronavirus disease 2019. Data are presented according to the American Heart Association’s 17-segment model. Data are presented as percentages.

All patients underwent CMR imaging after a median of 9 months (range 7–10 months) of their initial COVID-19 hospitalization. Their imaging results compared to the control group are reported in Table 2, and examples of CMR imaging are shown in Fig 2. Left ventricular dysfunction was present in five (5%) and right ventricular dysfunction in four (4%) COVID-19 patients. All individuals in the control group had normal left and right ventricular ejection fractions. None of the study participants had myocardial edema on CMR. Two COVID-19 patients refused gadolinium contrast agent injection at the time of imaging. Any detectable left ventricular LGE was more prevalent in subjects with a history of COVID-19 (66% vs. 37%, p<0.01). The prevalence of scar characteristics for myocardial infarction was comparable in both groups (8% vs. 2%, p = 0.18). Two of the eight COVID-19 patients with ischemic scars had a known history of myocardial infarction before their hospitalization. LGE suggestive of previous myocarditis was more common in COVID-19 patients than in the control group (29% vs. 9%, p = 0.01). The distribution of LGE in the study population is depicted in detail in Fig 3. No COVID-19 patient had a known reported history of myocarditis before COVID-19. No study participant had abnormal pericardial fluid, and three COVID-19 patients had pleural fluid on CMR.

**Table 2:** Results of CMR imaging during follow-up in patients with COVID-19 hospitalization and in the control group.

Variable	N	COVID-19 (n = 95)	Control (n = 43)	P-value
LVEDV (ml/m^2^)	138	77 (68–87)	82 (76–91)	0.02
LVEF (%)	138	60 (56–64)	59 (58–63)	1.00
LV mass (g/m^2^)	138	52 ± 11	52 ± 8	0.60
Left ventricular WMA	138	5	0	0.13
RVEDV (ml/m^2^)	138	79 ± 14	85 ± 15	0.03
RVEF (%)	138	58 (55–62)	59 (55–61)	0.93
Right ventricular WMA	138	2	0	0.34
Left atrial area (cm/m^2^)	138	11 ± 2	12 ± 2	0.03
Right atrial area (cm/m^2^)	138	10 (8–11)	12 (10–13)	<0.001
Left ventricular oedema	138	0 (0%)	0 (0%)	1.00
Abnormal rest perfusion	136	1 (1%)	0 (0%)	0.49
*Late gadolinium enhancement*				
Any LGE	136	61 (66%)	16 (37%)	<0.01
Ischemic pattern	136	7 (8%)	1 (2%)	0.13
Myocarditis pattern	136	27 (29%)	4 (9%)	0.01

Data are presented as number of patients (%) or mean ± SD, or median (interquartile range). LGE = late gadolinium enhancement; LV = left ventricular; LVEDV = left ventricular end-diastolic volume; LVEF = left ventricular ejection fraction; RVEDV = right ventricular end-diastolic volume; RVEF = right ventricular ejection fraction; WMA = wall motion abnormality.

### Associated risk factors for myocardial injury

Clinical, laboratory and CMR variables in COVID-19 patients with and without a myocarditis scar are shown in Table 3. In brief, as shown in Table 3, patients with a myocarditis scar had lower left ventricular ejection fraction, but only two patients had simultaneous left ventricular dysfunction (ejection fraction <50%). None of the other patient characteristics, including diabetes, hypertension, hypercholesterolemia, smoking, asthma, coronary artery disease, or atrial fibrillation, were related to the presence of myocarditis scar on CMR imaging (p-value always >0.05). Of all COVID-19 patients, two had elevated troponin-I levels at follow-up. One of them had a scar consistent with myocarditis, while the other had no LGE.

**Table 3:** Comparison of selected clinical and imaging variables in COVID-19 patients with and without myocarditis scar on follow-up.

Variable	N	Scar + (n = 27)	Scar–(n = 66)	P-value
*Clinical variables*				
Female	93	13 (48%)	36 (55%)	0.58
Age	93	58 ± 8	60 ± 11	0.19
Length of hospitalization (days)	93	11 (8–16)	5 (8–22)	0.17
COVID-19 vaccination	80	5 (20%)	11 (20%)	1.00
ICU treatment	93	14 (47%)	40 (67%)	0.44
*Laboratory variables at baseline*				
Troponin elevation	72	5 (22%)	10 (20%)	0.69
C-reactive protein (mg/l)	93	184 (108–246)	158 (108–235)	0.69
*Laboratory variables at follow-up*				
Hs-TnI (ng/l)	93	3 (3–5)	3 (3–5)	0.99
NT-ProBNP (ng/l)	93	53 (35–98)	73 (35–119)	0.48
Glomerular filtration rate (ml/min)	93	90 (83–99)	91 (81–98)	0.85
*Cardiac magnetic resonance imaging*				
LVEDV (ml/m^2^)	93	77 (68–89)	76 (68–85)	0.40
LVEF (%)	93	57 (55–61)	61 (57–64)	0.01
LV mass (g/m^2^)	93	53 ± 10	51 ± 11	0.33
RVEDV (ml/m^2^)	93	80 ± 15	79 ± 14	0.62
RVEF (%)	93	59 (55–62)	58 (55–62)	0.55

Data are presented as number of patients (%) or mean ± SD, or median (interquartile range). COVID-19 = coronavirus disease 2019; Hs-TnI = high-sensitivity troponin-I; LGE = late gadolinium enhancement; LV = left ventricular; LVEDV = left ventricular end-diastolic volume; LVEF = left ventricular ejection fraction; NT-ProBNP = N-terminal pro B*-*type natriuretic peptide; RVEDV = right ventricular end-diastolic volume; RVEF = right ventricular ejection fraction.

### Myocardial injury and long-term symptoms

**Fig 4:**
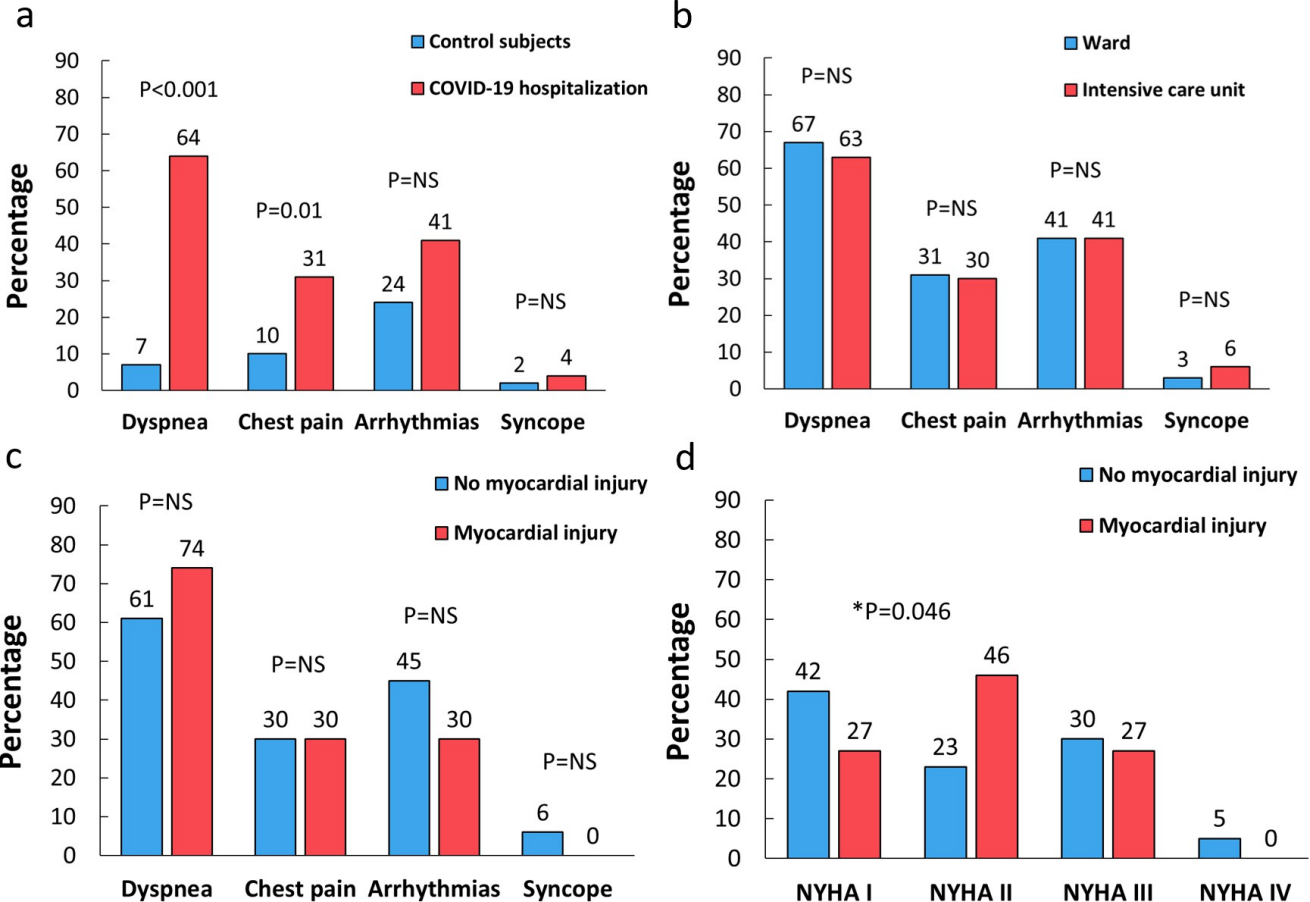
The Association of long-term symptoms with patient characteristics and cardiac magnetic resonance (CMR) imaging findings. Comparison of the symptomatic burden in patients with previous coronavirus disease 2019 (COVID-19) hospitalization and control subjects (**A**). Long-term symptoms in COVID-19 patients with and without intensive care unit treatment during hospitalization (**B**). The difference in symptomatic status in COVID-19 patients with and without a myocarditis scar on CMR imaging (**C**). New York Heart Association (NYHA) classification of dyspnea in patients with COVID-19 and a myocarditis scar on CMR (**D**).

Long-term symptoms in COVID-19 patients and in control subjects were assessed at a median of 9 months (range 7–10 months) after hospitalization at the time of CMR imaging. As shown in Fig 4, COVID-19 patients had more often dyspnea (63%) and chest pain (31%) than the control subjects. Previous ICU treatment or the presence of myocarditis scar were not related to higher symptomatic burden in COVID-19 patients. Similarly, self-reported severe dyspnea classified according to the NYHA (New York Heart Association) class at the time of CMR was similar in patients with myocarditis scar compared to other COVID-19 patients (Fig 4D). However, COVID-19 patients with myocarditis scar reported more often NYHA II grade dyspnea compared to other patients (Fig 4D). COVID-19 patients with any detectable LGE did not have a higher rate of dyspnea (66% vs. 63%, p = 0.77), chest pain (25% vs. 41%, p = 0.11), arrhythmias (30% vs. 66%, p<0.01) or syncope (5% vs. 3%, p = 0.66) at the follow-up. The presence of an ischemic scar on the CMR was not associated with an elevated risk for dyspnea (13% vs. 69%, p<0.01), chest pain (13% vs. 33%, p = 0.24), arrhythmias (13% vs. 43%, p = 0.09), or syncope (0% vs. 5%, p = 0.53) at follow-up. Patients with troponin elevation at the time of their initial hospitalization had comparable symptomatic burden with other COVID-19 patients regarding dyspnea (75% vs. 65%, p = 0.45), chest pain (31% vs. 32%, p = 0.98), arrhythmias (50% vs. 39%, p = 0.41) and syncope (7% vs. 4%, p = 0.60), respectively.

## Discussion

This prospective study demonstrates that left ventricular LGE on CMR is a frequent finding in patients previously hospitalized due to COVID-19. Interestingly, almost one-third of these patients had a myocardial scar suggestive of post-acute previous myocarditis, while the presence of an ischemic scar was less prevalent. The myocarditis scar was not related to sustained left ventricular dysfunction, ICU treatment in the acute phase, or higher symptomatic burden by the time of imaging. Moreover, none of the patients had signs of ongoing inflammation on CMR at follow-up. Thus, our results provide reassurance to clinicians since reported myocarditis-like LGE on CMR in COVID-19 patients seems to be mostly a subclinical finding that does not require further evaluation.

### Earlier CMR studies in brief

A few CMR studies predate our own with variable sample sizes and imaging times relative to the COVID-19 infection. As in our study, the non-ischemic LGE pattern suggestive of previous myocarditis has been common in COVID-19 patients. Two larger studies by Puntmann et al. and Kotetcha et al. had 100 and 148 patients with risk-factor matched controls and were conducted 2–3 months after COVID-19 [14, 15]. Puntmann et al. detected more ischemic (32%) and non-ischemic (20%) LGE compared to the control group in patients with mostly home-treated infection [14]. Similarly, Kotecha et al. demonstrated that subendocardial scars suggestive of myocarditis were prevalent in hospital-treated patients with troponin elevation (22%) [15]. Other forms of LGE were as common in the control subjects as in COVID-19 patients in their study. Huang et al. reported a high rate of abnormal CMR, defined as the presence of either LGE or elevated T2 time in 15 of their 26 symptomatic study patients at 1.5 months after hospitalization [16]. In other, smaller series of hospitalized patients with heterogenous study settings, the prevalence of non-ischemic scar suggestive of possible post-infection myocarditis has varied between 3–30% [17–20].

### Myocarditis scar and symptoms at follow-up

Our study explored the presence of dyspnea, chest pain, syncope, and arrhythmia symptoms after the initial COVID-19 hospitalization and their relation to CMR imaging findings. CMR is an excellent modality for evaluating the cardiac origin of these symptoms, their differential diagnosis, and risk stratification [9, 12]. It can differentiate ischemic from nonischemic disease in chest pain and dyspnea patients and detect left ventricular dysfunction [9, 12]. Myocarditis or ischemic scar can be an arrhythmogenic substrate for arrhythmia symptoms or syncope. Moreover, these differential diagnoses enable change in patient management through pharmacologic treatment of CAD and ventricular dysfunction, revascularization, and implantable device therapies for life-threatening arrhythmias.

Cardiovascular symptoms and decreased exercise tolerance have been common after COVID-19, which is also a risk factor for developing new cardiovascular diseases [5–7]. Although the long-term symptoms were common in our population, myocarditis scar was not associated with greater symptomatic burden or sustained left ventricular dysfunction. Furthermore, myocarditis scar was not associated with the need for ICU treatment during the initial hospitalization. Therefore, myocarditis scar after COVID-19 on cardiac imaging seems to be mostly subclinical in nature. In fact, the incidental myocarditis-like LGE was also detected in our control subjects, which suggests that some of the scars in COVID-19 patients might predate the SARS-CoV-2 infection. Nevertheless, detecting any myocardial scar might have clinical value as it might lower the threshold for the development of heart failure due to other causes and be an arrhythmogenic substrate.

We did not find signs of ongoing myocardial inflammation in our patients, and a troponin elevation at follow-up was rare. Thus, our study suggests that the clinical yield for CMR screening of unselected COVID-19 population for previous myocarditis would be low. In addition, a positive imaging finding may be unrelated to the clinical symptoms, which might be more commonly explained by other reasons, such as pulmonary sequelae of COVID-19. Sustained troponin elevation or depressed left ventricular function may be more appropriate indications for CMR imaging according to standard clinical practice, and the possibility of COVID-19-induced autoimmune diseases such as cardiac sarcoidosis should not be forgotten [21].

#### Limitations

It should be noted that our study population underwent CMR at a post-acute phase, and we were unable to diagnose myocarditis according to the prevailing Lake-Louise criteria [22]. However, we aimed to study the clinical yield of CMR in the chronic phase of COVID-19 and not in at acute setting. Thus, our results do not prove that the myocarditis scars were consequence of COVID-19. The LGE suggestive of previous myocarditis may have existed before or developed after the COVID-19. A subset of the participants received COVID-19 vaccination before CMR imaging, which is an unlikely, but possible, cause of myocarditis in the older age group [23]. There is a potential referral bias as patients who died due to COVID-19 were not included. We did not recruit any pediatric patients or patients with mild infection. However, our study patients with ongoing symptoms are well representative of the previously severely ill adult patients seen at outpatient clinics after initial COVID-19 hospitalization. Detection of LGE in the thin inferolateral myocardium can be challenging. Our imaging results were verified by a level 3 accredited CMR imager to optimize the accuracy and generalizability of our findings. Invasive angiography or other coronary imaging was not performed at follow-up. Symptoms at follow-up were screened by a questionnaire, which is subjective. More objective measures for symptomatic burden and exercise tolerance such as cardiopulmonary exercise testing might have been valuable.

## Conclusions

Myocardial scar suggestive of previous myocarditis was detected in one-third of hospital-treated COVID-19 patients, but it was not associated with greater symptomatic burden, need for ICU treatment, sustained ventricular dysfunction or active myocardial edema at follow-up. Thus, myocarditis-like LGE on CMR after COVID-19 hospitalization seems to be of little clinical significance.

## Supporting information

S1 Checklist STROBE checklist for an observational study. (PDF)

S1 Protocol Original study protocol written in Finnish. (PDF)

S2 Protocol Original study protocol translated to English. (DOCX)

S1 Dataset Minimal dataset of the manuscript. (XLSX)
