# Supplementary material for: Cardiac magnetic resonance -detected myocardial injury is not associated with long-term symptoms in patients hospitalized due to COVID-19
Source: PLoS One. 2023 Mar 8;18(3):e0282394. doi: 10.1371/journal.pone.0282394 (PMC9994679; doi:10.1371/journal.pone.0282394)
Supplement: S1 Protocol — (PDF) [file pone.0282394.s002.pdf]

Tutkimussuunnitelma: Toipuminen kriittisestä koronavirusinfektiosta

I Taustaa

Tehohoidon jälkeinen pitkäaikaisselviytyminen on vasta viime vuosina noussut alan tutkimuksessa keskeiseen asemaan. Potilaan palautuminen kriittisestä sairaudesta kestää pitkään, ja sairaalasta elossa kotiutuneiden kuolemanriski on pitkään lisääntynyt. Sen lisäksi potilaille voi jäädä kriittisestä sairaudesta, sen hoitomenetelmistä ja pitkästä sairaalassa olosta monia elämän laatua heikentäviä oireita.
Tehohoidon jälkeen on kuvattu jopa nuorilla ja aiemmin terveillä keskivaikeaa Alzheimerin tautia vastaavaa kognitiivisten toimintojen heikkenemistä[1]. Kaikkia syitä siihen ei tiedetä, mutta esimerkiksi tehohoidon aikainen varsin yleinen (jopa 80%) sekavuus eli delirium, on tunnistettu riskitekijä[1,2]. Meneillään olevassa tutkimusprojektissamme ASSESS-shock 2:ssa (TYH2020341) selvitämme verenkiertovajauksen yhteyttä deliriumiin ja neuropsykologiseen toipumiseen sekä tehohoidon aikaisiin muutoksiin elimistön mikrobiomissa.
Koronaviruksen aiheuttama COVID-19 on aiheuttanut merkittävän tehohoidon tarpeen lisääntymisen myös työikäisten keskuudessa. Pitkäaikaisseurannassa ilmenevistä haitoista ei COVID-19-pneumoniitin osalta vielä ole tietoa, mutta vaikean hengitysvajauksen (ARDS, acute respiratory distress syndrome) jälkeen tiedetään esiintyvän pitkäaikaista rasituksessa ilmenevää hengenahdistusta, ja kognitiivisen suorituskyvyn heikkenemistä vielä yhden ja jopa useankin vuoden kuluttua sairaalahoidosta. [3,4,5] COVID-19-potilaista kertyneen oman kliinisen kokemuksen ja kirjallisuuden perusteella vaikean hengitysvajauksen lisäksi COVID-19-potilailla esiintyy huomattavan paljon neurologisia komplikaatioita [6,7]. Deliriumin esiintyminen on erittäin yleistä ja pitkäkestoista; sille altistaa todennäköisesti raskas pitkäkestoinen hengityslaitehoito ja sen edellyttämä anesteettien ja opioidien pitkäkestoinen käyttö. On myös mahdollista, että koronavirus vaikuttaa suoraan keskushermostoon ja lisää useiden muidenkin neurologisten komplikaatioiden kuten kouristusten, enkefaliitin, aivoverenkiertohäiriöiden ja polyradikuliitin riskiä[8,9].

Aivovaurion arvioimiseksi on tutkimuskäytössä uusia laboratoriomerkkiaineita. Neurofilamentti (kevyt) (neurofilament light, NfL) on intra-aksonaalinen proteiini, jota tihkuu ulos degeneroituvista aksoneista. Tämä lisää NfL konsentraatiota aivo-selkäydinnesteessä ja veressä potilailla, joilla on keskushermoston sairaus, johon liittyy neuronien vaurio. Aivo-selkäydinnesteen ja veren pitoisuudet korreloivat hyvin eri neurologisissa sairaustiloissa keskenään ja sairauden vaikeusasteen kanssa. Tämä viimeksi mainittu on osoitettu neurodegeneratiivisissa sairauksissa, mutta myös äskettäin akuutissa hypoksis-iskeemisessä vauriossa sydänpysähdyspotilailla.[10] Neurofilamentin pitoisuus nousee hitaasti, viikkojen kuluessa ja säilyy poikkeavana kuukausia. NfL:a pidetään tällä hetkellä parhaana neurodegeneraation merkkiaineena, koska sillä on vain vähän sekoittavia virhelähteitä. [10, 11] Plasman NfL:n on todettu nousevan liittyen akuuttiin neurologiseen vaurioon myös akuuttivaiheen Covid-19:ssa.[12]

ApoE-lipoproteiini on elimistön rasva-aineenvaihduntaan osallistuva lipoproteiini, joka on lisäksi yhteydessä hypertension ja ateroskleroosin riskiin sekä deliriumin ja neurodegeneratiivisen sairauden kuten Alzheimerin taudin riskiin.[13] Sitä on kolmea alleelityyppiä ApoE2, ApoE3 ja ApoE4, jotka esiintyvät kuutena erilaisena kombinaationa. Eurooppalaisessa väestössä ApoE4-alleelia on n 15-20%:lla väestöstä ja sen suhteen

homotsygooteilla on 14-kertainen riski sairastua Alzheimerin tautiin. ApoE4:llä on osoitettu *in vitro* olevan yhteys tiettyjen virustyyppien helpompaan pääsyyn soluun. Äskettäin julkaistussa biopankkiaineistoon perustuvassa tutkimuksessa todettiin, että ApoE4-alleeli näytti altistavan potilaita vaikeammalle Covid-19 taudinkuvalle, kun siihen assosioituvat perussairaudet huomioitiin analyysissa.[14] Yhteydestä neurologisiin komplikaatioihin Covid-19 akuutissa vaiheessa tai myöhäiskomplikaatioina akuutin sairauden jälkeen ei tietääksemme ole aikaisempia tutkimuksia.

Sydänentsyymien on raportoitu kohoavan noin 12-35 %:lla sairaalahoidetuista Covid-potilaista ja etenkin vaikeassa taudinkuvassa, oletettavasti infektioon liittyvän sydänlihastulehduksen (myokardiitin), kammiokuormituksen tai iskemian pohjalta.[15, 16] Myokardiitti voi olla oirekuvaltaan oireeton, esiintyä rytmihäiriöinä tai etenevänä vajaatoiminta ja kardiogeenisena shokkina.[17] Covid-infektion pitkäaikaisvaikutuksista sydänterveyteen, kuten infektion jälkeisistä arvista ja sydämen toimintakyvystä, ei ole toistaiseksi tutkittua tietoa. Myös Covid-infektion vaikea keuhkoaffisio ja taipumus keuhkoveritulppiin voi kuormittaa erityisesti sydämen oikeaa kammiota.[18]
Veren hyytymistaipumus on lisääntynyt Covid-19-potilailla, ja jo pandemian alkuvaiheessa hyytymisjärjestelmän aktivaatiolla todettiin olevan yhteys kuolleisuuteen, ja sittemmin sairauden akuuttivaiheessa mm koholla olevan D-dimeerin on osoitettu olevan voimakkaasti yhteydessä kohonneeseen kuoleman riskiin.[19, 20] Hyytymisjärjestelmän palautumisesta pidempiaikaisessa seurannassa ei tietääksemme ole vielä kattavasti tutkimustietoa.

Tehohoidon jälkipoliklinikalla on jo kymmenen vuoden ajan seurattu potilaiden toipumista varsinkin pitkien tehohoitojaksojen jälkeen ja käyty potilaiden kanssa läpi tehohoidon aikaisia kokemuksia. Vielä julkaisemattoman aineistomme perusteella 2-3 kuukautta tehohoidon jälkeen potilaat raportoivat edelleen kärsivänsä monista elämän laatuun vaikuttavista oireista, kuten unettomuus, painajaiset, masennus, haluttomuus, uupumus ja kognitiiviset ongelmat. Muistikuvat deliriumista ovat yleisiä ja häiritseviä ja niiden läpikäyminen potilaiden kanssa on keskeisen tärkeää ja jo niiden sanoittaminen ja normaaliksi toteaminen auttaa monia. Tällä hetkellä tehohoidon jälkipoliklinikka on tilapäisesti tauolla liittyen tietojärjestelmämuutoksen tuomiin haasteisiin. COVID-potilaat uhkaavat siis jäädä vaille oleellisen tärkeää jälkitapaamista tehohoitolääkärin kanssa. Tästä syystä haluamme nyt tutkimushankkeena järjestää jälkipoliklinikkakäynnin, jossa potilaan kanssa käydään läpi tehohoitoon ja hänen nykyiseen vointiinsa liittyviä asioita ja samalla tutkitaan tehohoidon pitkäaikaistoipumiseen liittyen neurologista ja neuropsykologista toipumista kliinisin testein ja kuvantamisen keinoin. Poliklinikkakäynnillä tutkitaan myös fyysisen suorituskyvyn, hengityselimistön ja immuunipuolustuksen palautumista vaikeasta sairaudesta sairaudesta ja selvitetään taudin aikana tai sen jälkeen ilmaantuneita sydänoireita.

Hajut herättävät voimakkaita sekä positiivisia että negatiivisia tuntemuksia. Niinpä hajuaistin menetys voi merkitsevästi huonontaa elämänlaatua. Viruksen aiheuttama ylähengitystietulehdus on yleisimpiä syitä hajuaistin menetykseen. COVID-19-pandemian aikana koronavirusinfektioon (SARS-CoV-2) sairastuneilla osalla haju- ja makuaistin muutokset ovat olleet jopa ensioireena (Whitcroft and Hummel 2020).[21]
Ylähengitystieinfektion jälkeisestä hajuaistin häiriöstä toipuu spontaanisti 32-66%.[22]
Hajuharjoittelun on osoitettu auttavan hajuaistin toipumisessa.[23] Hajukäämin koolla on osoitettu olevan ennusarvoa hajuaistin toipumisen suhteen.[24] Koronavirusinfektiossa

osalla potilaista hajuaisti on korjaantunut kahdessa viikossa. Pitkäaikaisseurantoja hajuaistin toipumisen suhteen ei vielä ole tiedossa.

Tutkimuksessa halutaan myös selvittää voiko hyperinflammatorisen tilan jatkuminen liittyä pitkittyneisiin oireisiin Covid-19 jälkeen. Matrix metalloproteinaasien (MMP) -7 ja -9 on kuvattu esiintyvän kohonneina pitoisuuksina monissa kroonisissa keuhkosairauksissa ja äskettäin myös MMP-9:n osoitettiin assosioituvan vaikeampaan Covid-19 hengitysvajaukseen.[25] MMPt ovat solun välitilan muokkaukseen osallistuvia mm neutrofiilisista granulosyyteista vapautuvia endopeptidaaseja, joilla on moninaisia rooleja akuutissa ja kroonisessa inflammaatiossa. MMP-8 –tasot mm assosioituvat reaktiiviseen artriittiin ja hyytymishäiriöihin.[26, 27] MMP säätelyyn osallistuvat mm proinflammatoriset sytokiinit ja kudostasolla niitä säätelevät MMP kudosinhibiittorit TIMP:t. TIMP-1 on kuvattu assosioituvan itsenäisenä ennustetekijänä akuutin hengitysvajauspotilaan 1 vuoden kuolleisuuteen. [28]

II Tutkimuksen kysymyksenasettelu, hypoteesi, tutkimuksen tavoite

Tutkimuksen tavoite on jälkipoliklinikkatapaamisen ohella selvittää (K=kysymyksen asettelu, H=hypoteesi):

1) K: Esiintyykö koronaviruksen takia teho-osastolla hoidetuilla potilailla laajoissa neuropsykologisissa testeissä esiin tulevaa kognitiivisen suorituskyvyn heikkenemistä yleisväestöstä määritettyihin viitearvoihin verrattuna?
   H: Neuropsykologisissa testeissä havaitaan osalla potilaista muutoksia verrattuna saman ikäiseen yleisväestöön
2) K: Esiintyikö tehohoidon aikana kliinisesti havaittuja neurologisia komplikaatioita
   H: Tehohoidon aikana esiintyi neurologisia komplikaatioita kuten deliriumia ja aivoverenkiertohäiriöitä.
3) Miten potilaat ovat palautuneet vaikeasta sairaudesta neurologisesti? H: Suurin osa on toipunut neurologisesti hyvin, mutta osalla saattaa löytyä tarkassa tutkimuksessa oireita.
4) K: Näkyykö MRI-tutkimuksessa muutoksia verrattuna vuodeosastolla hoidettuihin Covid-19-potilaisiin, jotka selviytyivät ilman tehohoitoa.
   H: Potilailla, joilla esiintyy kliinisessä tutkimuksessa neurologisia poikkeavuuksia voi löytyä radiologisesti havaittavia muutoksia pään MRI-tutkimuksessa.
5) K: Onko potilaiden neurologisen oireisto yhteydessä valkean aineen vaurioon, joka voitaisiin havaita määrittämällä neurofilamenttitasoja? H: Potilailla, joilla on edelleen neurologisia tai neurokognitiivisia oireita, voidaan havaita plasman neurofilamenttitasossa verrattuna non-Covid potilaisiin ja Covid-potilaisiin, jotka ovat neurologisesti oireettomia.
6) K: Onko ApoE4-alleeli yleisempi potilailla, joilla on vaikea Covid-19-taudinkuva?
   H: Tehohoitoon ja sairaalahoitoon joutuneilla Covid-19-potilailla on useammin todettavissa yksi tai kaksi ApoE4-alleelia kuin kotona Covid-19:n sairastaneilla potilailla tai verrokeilla, jotka eivät ole sairastaneet koronavirusinfektiota.

7) Ennustavatko koholla olevat neurofilamenttitasot ja jokin ApoE-alleeli pitkäaikaisia neurologisia oireita? H: Potilailla, joilla on koholla oleva neurofilamenttitaso 6kk sairaudesta on enemmän neuropsykologisia muutoksia 5v:n kuluttua sairaudesta.

8) K: Onko potilailla haju- tai makuaistin heikkenemistä verrattuna normaaliväestöön 6kk sairastumisesta ja onko hajuaistin neuraalisten ratojen alueella nähtävissä muutoksia MRI-tutkimuksella?
H: Osalla potilaista esiintyy haju- ja/tai makuaistissa muutoksia, jotka ovat huonosti tai vain osittain palautuneet 6kk sairastumisesta. MRI-tutkimuksessa hajuhermon tai neuraalisen radan alueella nähdään muutoksia.
9) K: Millainen on potilaiden fyysinen suorituskyky ja keuhkojen funktiota kuvaavat parametrit 3 ja 6 kuukautta ja 2 vuotta tehohoidon jälkeen? H: Osalla potilaista keuhkofunktio ja fyysinen suorituskyky eivät ole palautuneet saman ikäisestä normaalipopulaatiosta laskettujen viitearvojen tasolle.
10) K: Minkälainen on potilaiden immuunijärjestelmän tila (esiintyykö pitkäkestoista anergiaa, tai muita leukosyyttien toimintahäiriöitä 6kk tehohoidon jälkeen)? H: Immuunijärjestelmän toimintakyky saattaa olla edelleen heikentynyt 6kk sairauden jälkeen.
11) K: Onko teho- tai vuodeosastohoidossa olleilla tai kotona koronavirusinfektion sairastaneilla henkilöillä koronavirusinfektioon liittynyt ohittuneita tai jatkuvia sydänoireita? H: Koronavirusinfektion aikana ja/tai sen jälkeen on esiintynyt sydänoireita ja ne ovat olleet yleisempiä ja jatkuneet pidempään tehohoitoa vaatineilla potilailla.
12) K: Näkyykö sydämen magneettikuvauksessa myokardiittimuutoksia ja ovatko ne yhteydessä potilaiden heikompaan toipumiseen ja sydänoireiluun. H: Joidenkin, etenkin tehohoitoa tarvinneiden potilaiden väsymyksen ja suorituskyvyn hitaan palautumisen taustalla voi olla magneettikuvauksella löydettävissä oleva sydänlihastulehduksen jälkitila.
13) K: Onko veren hyytymistekijöissä muutoksia nähtävissä Covid-19-infektion sairastaneilla potilailla 6kk akuutin sairastamisvaiheen jälkeen verrattuna akuuttivaiheessa otettuihin kliinisiin hyytymistekijänäytteisiin? H: Veren hyytymistekijät ovat normalisoituneet 6kk sairastumisen jälkeen.

14) SARS-CoV-2 immuniteettia selvittävän osatutkimuksen tavoitteena on kehittää testiä Covid-19 immuniteetin mittaukseen ja tuoda lisätietoa infektioon liittyvästä immuniteetistä ja sen kestosta. Tämä on tärkeää myös Covid-19 rokotteen tehokkuuden seurannassa, sillä testillä voidaan mitata erikseen rokotteen aikaansaamaa ja infektiosta johtuvaa immuunivastetta.

15) K: Onko 6kk akuutin sairastumisen jälkeen havaittavissa kohonneita MMP -7, -8, tai -9-pitoisuuksia tai niiden säätelyyn osallistuvien sytokiinien tai kudosinhibiittorien poikkeavia pitoisuuksia ja assosioituvatko mahdollisesti koholla olevat tasot kliinisiin oireisiin ja löydöksiin mm keuhkofunktiossa tai kuvantamisessa? H: Osalla potilaista, jotka oireilevat edelleen voimakkaasti, on nähtävissä koholla olevia tulehdusmarkkereita.

Kaiken kaikkiaan tavoitteena on karakterisoida siis potilaiden pitkäaikaistoipumista ja tarvittaessa ohjata potilaat asianmukaisiin jatkoselvityksiin tai kuntoutukseen.

III Tutkimussuunnitelma, menetelmät ja toteuttaminen

Tutkimusaineistona ovat HUS-alueen teho-osastoilla koronavirusinfektion vuoksi hoidetut aikuiset potilaat, jotka täyttävät sisäänottokriteerit ja joilla ei ole tutkimuksen vasta-aiheita.

Sisäänottokriteerit:
-18 vuotta täyttänyt
- asiointikieli on suomi tai ruotsi. Tutkimuksen neuropsykologiseen osatutkimukseen voidaan ottaa mukaan ainoastaan henkilöitä, joiden asiointikieli on suomi, koska testeistä ei ole suomenruotsalaisille validoituja versioita ja testien tulokset ovat hyvin kielisensitiivisiä. Kaikkiin muihin osatutkimuksiin voidaan ottaa mukaan myös henkilöitä, joiden asiointikieli on ruotsi.
-ollut tehohoidossa jollakin HUS:n teho-osastoista vaikean koronavirusinfektion takia
-on mukana RECOVID-tutkimuksessa

Poissulkukriteerit:
-raskaus
-ennen koronavirusinfektiota diagnosoitu merkittävä neurologinen sairaus, kuten vaikean aivovamman tai aivoverenkiertohäiriön jälkitila
-aistivamma
-muistisairaus
-Parkinsonin tauti
-kehitysvamma

Osatutkimuskohtaiset poissulkukriteerit, mikäli niitä on, luetellaan kunkin tutkimusosion kuvauksen yhteydessä. Oletettu potilasmäärä on n 100-150. Potilaat identifioidaan tehohoidon jälkeen rekisteröidyn Covid-19-diagnoosin avulla tehohoidon laatutietokannasta, minkä jälkeen heihin otetaan yhteyttä ja pyydetään osallistumaan tutkimukseen. Potilaille lähetetään potilaan ja lähiomaisen tutkimustiedote ja suostumuslomake, jossa on tutkimusryhmän yhteystiedot lisätietoja varten. Tutkimuksen jatko-osaa varten tutkittaville lähetetään uudet tutkittavan tiedotteet ja suostumuslomakkeet postitse tai heidän suostumustaan kysytään tavattaessa heidät jälkipoliklinikalla. Tutkittavien joukossa on vain suomea asiointikielenään käyttäviä henkilöitä, joten tutkimusasiakirjat on jatkotutkimuksen osalta laadittu vain suomen kielellä.

Verrokkipotilaita rekrytoidaan lehti-ilmoituksella ja sosiaalisen median avulla. Verrokkipotilaiksi soveltuvat varmistetun koronavirusinfektion sairastaneet henkilöt, joilla sairauden vaikeusaste ei ole edellyttänyt tehohoitoa, vaan infektio on sairastettu kotona (N=50). Toinen verrokkiryhmä, potilaat, jotka saivat hoitoa koronavirusinfektioon sairaalan vuodeosastolla (N=50), identifioidaan suostumuksen kysymistä varten niistä potilaista, jotka saavat/ovat saamassa kutsun keuhkosairauksien klinikan järjestämään jälkikontrolliin. Verrokkihenkilöiltä pyydetään erillinen kirjallinen suostumus tutkimukseen osallistumiseen.

Rekrytoimme myös 50 verrokkihenkilöä, jotka eivät ole sairastaneet Covid-19-infektiota. Taulukossa alla näkyvät ne tutkimuksen osatutkimukset, joissa näitä ns non-Covid-verrokkeja tullaan vertaamaan muihin tutkittaviin. Non-Covid-verrokit pyritään löytämään 14.9.2020 mennessä suostumuksensa tutkimukseen antaneiden tutkittavien lähipiiristä, jotta he olisivat sosioekonomisen statuksen ja koulutustason suhteen mahdollisimman samankaltaisia kuin tutkittavat teho-osastolla hoidetut henkilöt. Käytännössä tutkittavilta

kysytään jälkipoliklinikalla, onko heidän lähipiirissään henkilöitä, jotka eivät ole sairastaneet Covid-19:a ja joiden he arvioisivat olevan halukkaita osallistumaan tutkimukseen. Heille välitetään tutkimusryhmän yhteystiedot, jotta he voivat halutessaan ilmoittaa kiinnostuksensa tutkimusta kohtaan. Tämän jälkeen mahdollisille non-Covid-verrokkihenkilöille lähetetään tutkittavan tiedote ja suostumuslomake palautuskuorineen. Jos tätä kautta ei löydetä riittävästi non-Covid verrokkeja, voidaan heitä etsiä myös Helsingin Yliopiston henkilökunnasta eri ammattiryhmistä ilmoittamalla rekrytoinnista Yliopiston intranet-sivuilla. Verrokkeja pyritään saamaan eri ikä- ja ammattiryhmistä siten, että koulutustaso ei eroa merkittävästi tutkittavien (tehohoidossa Covidin vuoksi olleet) koulutustasosta.

Potilaat, jotka antavat suostumuksensa, saavat kutsun saapua tutkimus- ja poliklinikkakäynneille 6 kuukautta tehohoidon jälkeen. Ennen poliklinikkakäyntiä 3kk sairaudesta tutkimuspotilaille tehdään puhelimitse lyhyt neurologinen ja neuropsykologinen kartoitus ja heille postitetaan kaksi kyselyä. Laajemmat kyselyt tehdään kahdella erillisellä käynnillä 6kk tehohoidosta. Oheisessa taulukossa on esitetty aikapisteet ja niihin suunnitellut kartoitukset. Uppsalan Akateemisessa sairaalassa ja Oslon Yliopistosairaalassa (mahdollisesti) tehdään osa taulukosta mainittuja testejä vastaaville potilasryhmille ja niiden osalta aineistot yhdistetään. Uppsalassa tehdään mRS, RAND-36, MOCA ja IQCODE-testit.
MRI-tutkimuksen verrokkipotilaita etsitään lehti-ilmoituksen avulla, minkä jälkeen yhteydenoton perusteella heiltä pyydetään tietoon perustuva suostumus osallistumisesta tutkimukseen.

Taulukko 1. Tutkimuksessa käytettävät testit, työnjako, aikapisteet ja tutkittavien lukumäärä HUS:ssa.*=Erillinen suostumus

Helsinki			
**3 kk**	**Kuka tekee**	**Potilasmäärä**	**Verrokit**
Kliinisten tietojen rekisteröinti potilasasiakirjoista (neurologiset oireet ja tutkimukset tehohoidon aikana, delirium, hoidon kesto ja toimenpiteet, taustatiedot)	Johanna Hästbacka<br>Henriikka Ollila<br>Salla Kattainen<br>Minna Tallgren<br>Erika Wilkman	100-150	50 vuodeosastohoidossa ollutta

Puhelinhaastattelu: modified Rankin Scale (mRS)	Marjaana Tiainen<br>Laura Hokkanen<br>Sanna Koskinen<br>Johanna Hästbacka<br>Henriikka Ollila	100-150	50 vuodeosastohoidossa ollutta+ 50 kotona sairastanutta Covid-potilasta
Puhelinhaastattelu: Montreal Cognitive Assessment (T-MoCA)	-"-	100-150	50 vuodeosastohoidossa ollutta+ 50 kotona sairastanutta Covid-potilasta<br>50 non-Covid verrokkia
Behavior Rating Inventory of Executive Function, adult version (BRIEF-A) +BRIEF informant version<br>ABNAS Neuropsychological questionnaire	postitse	100-150	,,
**6 kk**			
RAND-36	postitse	100-150	50 vuodeosastohoidossa ollutta+ 50 kotona sairastanutta Covid-potilasta<br>50 non-Covid verrokkia
MFI-20	postitse	100-150	,,
HADS	postitse	100-150	,,
Informant Questionnaire on Cognitive Decline (IQCODE- CA)	postitse	100-150	50 vuodeosastohoidossa ollutta+ 50 kotona sairastanutta Covid-potilasta
Behavior Rating Inventory of Executive Function, adult version (BRIEF-A) +BRIEF informant version	Postitse (tai nps tutkimuksen yhteydessä)	100-150	50 vuodeosastohoidossa ollutta+ 50 kotona sairastanutta Covid-potilasta<br>50 non-Covid verrokkia
Taustatiedot	Pkl-käynnillä (ks Käynti 1 alla)	100-150	,,
modified Rankin Scale (mRS)	Pkl-käynnillä	100-150	,,
Montreal Cognitive Assessment (MoCA)	Pkl-käynnillä	100-150	,,
National Institute of Health Stroke Score (NIHSS)	Pkl-käynnillä	100-150	,,
CFS-9 (clinical frailty scale)	Pkl-käynnillä	100-150	,,

Hajuaistin seulontatesti	PKl-käynnillä	100-150	„
Keuhkosairauksien jälkiseuranta (Potilaan suostumuksella tiedot retrospektiivisesti vertailuun)	Keuhkojen rtg-kuva, spirometria ja diffuusiokapasiteetin mittaus, puhelinhaastattelu (iho- ja allergiasairaala)	100-150	50 vuodeosastohoidossa ollutta kliinisten keuhkofunktiotutkimusten tiedot
**Käynti 1**			
Neuropsykologinen tutkimus Haartmaninkatu 3	Opiskelija /Laura Hokkanen/Sanna Koskinen/ Riikka Pihlaja/ Annamari Tuulio-Henriksson	100-150	50 vuodeosastohoidossa ollutta+ 50 kotona sairastanutta Covid-potilasta+ 50 non-Covid verrokkia
Laboratorionäytteet (immunologia)	HUSLAB	10-50	
Aivojen MRI (ULS)	HUS kuvantaminen Anne Komsi, Juha Martola, Linda Kuusela	50 tehohoidossa ollutta (vähintään 30)	50 vuodeosastohoidossa ollutta+ 50 kotona sairastanutta Covid-potilasta<br>50 non-Covid verrokkia
Hajuaistin ja makuaistin testaus	Tutkimushoitaja	50 tehohoidossa ollutta	-”-
**Käynti 2**			
6 minuutin kävelytesti<br><br>Spirometria ja diffuusiokapasiteetti	Tutkimushoitaja, fysioterapeutti Linda Ulenius, erl Salla Kattainen, LK Inari Taskila (6mwt)<br><br>Kliininen fysiologia Päivi Piirilä	100-150	
Tehohoitolääkärin tapaaminen, tulosten kertominen ja keskustelu mahdollisista jatkosuunnitelmista	Johanna Hästbacka/Salla Kattainen/Henriikka Ollila	100-150	
Laboratorionäytteet Fibroosin/inflammaation merkkiaineet<br><br>Neurofilamentti +<br><br>ApoE-alleelit*	MMP ja TIMP-1 analyysit Mari Hämäläisen TAU<br><br>Kaj Blennow, Henrik Zetterberg (Göteborgin Yliopisto)<br>Liisa Myllykangas	100-150	50 vuodeosastohoidossa ollutta+ 50 kotona sairastanutta Covid-potilasta+<br>50 non-Covid verrokkia

Vasta-ainemääritysten menetelmätestaus<br><br>Hyyttek,<br>CAT= kalibroitu automoitu trombogrammi, mikrovesikkelit, PF4	Vilja Pietiäinen<br>Jussi Hepo-oja<br>Olli Vapalahti<br><br>Riitta Lassila<br>Mirka Sivula<br>Lotta Joutsi-Korhonen (HUSLAB)<br>Tuukka Helin (HUSLAB)		
**6-12 kk**			
Sydämen magneettikuvaus	HUS-kuvantaminen Valtteri Uusitalo, Suvi Syväranta, Satu Vaara, Sari Kivistö, Miia Holmström	50 tehohoidossa ollutta<br>50 vuodeosastohoidossa ollutta	50 non-Covid verrokkia
Sydäntutkimuksen laboratorionäytteet P-TnI, Nt-BNP, PVK, krea,, EKG	HUSLAB	50 tehohoidossa ollutta<br>50 vuodeosastohoidossa ollutta	50 non-Covid verrokkia
**12kk**			
Vasta-ainemääritysten seurantanäytteet		100-150	50 vuodeosastohoidossa ollutta+ 50 kotona sairastanutta Covid-potilasta
**24kk**			
Vasta-ainemääritysten seurantanäytteet		100-150	50 vuodeosastohoidossa ollutta+ 50 kotona sairastanutta Covid-potilasta
6MWT	fysioterapeutti Linda Ulenius	100-150 tehohoitopotilasta	
**5v**			
Poliklinikkakäynti ja neuropsykologinen testaus	Tehoklinikan tutkijat, Johanna Hästbacka, TBA	100-150	50 vuodeosastohoidossa ollutta+ 50 kotona sairastanutta Covid-potilasta<br>50 non-Covid verrokkia

Kuvantaminen

Aivojen magneettikuvissa arvioidaan neuroradiologin toimesta näkyviä muutoksia aivorakenteissa (esim. infarkteja ja verenvuotoja). Neuroradiologi Juha Martola suunnittelee tutkimukseen soveltuvan kuvantamisprotokollan fyysikko Linda Kuuselan kanssa ja ohjeistaa kuvantamisen logistiikasta vastaavaa hoitajaa Anne Komsia ja vastaa MRI-kuvantamisen tulosten tulkinnasta.
Lisäksi fyysikko Linda Kuuselan toimesta jatkotyöstetään kuvamateriaalia Meilahden fyysikoiden kuvalaboratoriossa tietokoneohjelmilla (esim. Slicer ja Freesurfer), jolloin pyritään havainnollistamaan esim. hajuhermon vauriota, hermoratojen vaurioita ja harmaan/valkea aineen atrofiaa.
Jotta kuvantamislöydöksiä voitaisiin verrata neuropsykologisissa testeissä saatuihin tuloksiin, tulee ainakin 30 MRI-kuvantamiseen osallistuvan potilaan kuulua myös neuropsykologiseen osatutkimukseen, ts heidän asiointikielensä tulisi olla suomi.
Mikäli aivojen magneettikuvantamisessa todetaan sattumalöydöksiä, kuten aivovaltimoaneurysmia tai muita potentiaalisesti kliinisesti tärkeitä löydöksiä, Juha Martola ottaa yhteyttä tutkimuksesta vastaavaan henkilöön, joka yhdessä neurologi Marjaana Tiaisen kanssa järjestää tarvittavat jatkoselvittelyt ja lähetteen adekvaattiin hoitoon.
Vasta-aiheet: Vaikea ahtaan paikan kammo, sydämen tahdistin, metalliset vierasesineet kehossa tai muu tunnettu MRI-tutkimuksen vasta-aihe. Tarkempi vasta-aiheiden kartoitus tehdään HUS ohjeen mukaisesti ennen kuvantamista.

Sydämen magneettikuvaus ajoittuu aikavälille 6-12kk sairaalahoidon päättymisestä.
Sydämen magneettikuvauksista vastaavat dosentit, sydänradiologit Sari Kivistö ja Miia Holmström yhdessä sydänradiologiaan suuntautuvien HUS-kuvantamisen erikoistuvien lääkäreiden LT Valtteri Uusitalon, LT Suvi Syvärannan ja LT Satu Vaaran kanssa.
Kuvaus suoritetaan HUS-Kuvantaminen radiologian sydänkuvantamisyksikön kliinisen sydänlihastulehduksen kuvantamisprotokollan mukaisesti 1.5 Teslan laitteella. Molempien kammioiden tilavuudet ja ejektiofraktio mitataan lyhyen akselin liikekuvien volumetriaan perustuen. Lisäksi mitataan pitkittäissuuntaista ja lyhyen akselin suuntaista liikekuvista paikallista sydänlihaksen liikettä strain-menetelmillä.[29] Sydänlihaksen ödeemaa ja sidekudoslisää mitataan kvantitatiivisesti T1- ja T2-painotteisista relaksaatioaikakartoista .[30] Gadoliniumtehosteisista jälkitehostumakuvista määritetään mahdollisen arven lokalisaatio ja prosentuaalinen koko suhteessa koko vasemman kammion massaan.
Tutkittaville, joilla on osastohoidon aikainen troponiinipäästö tai aiemmin toteamattomalle sydäninfarktille sopiva sydänlihasarpi, tehdään kardiologin kliinisen harkinnan mukaan sepelvaltimoiden tietokonetomografiakuvaus (TT). Mikäli sepelvaltimotilanne on kuitenkin arvioitavissa aiemmista tutkimuksista, kuvausta ei tehdä. Sepelvaltimoista arvioidaan hemodynaamiseti merkittävien sepelvaltimoplakkien määrää, ateroskleroosin laaja-alaisuutta ja mahdollisia sepelvaltimoiden aneurysmia, jollaisia on liitetty koronavirusinfektioon lapsilla ja nuorilla.[31] Mikäli muutoksia todetaan, dosentti, kardiologian erikoislääkäri Tiina Heliö ohjelmoi tarvittavat jatkotutkimukset.
Poissulkukriteerit sydämen magneettikuvaukseen: Pysyvä sydämen tahdistin tai jo ennen sairastumista todettu vaikea-asteinen iskeeminen tai muu sydänlihassairaus.
Vasta-aiheet: Vaikea ahtaan paikan kammo, varjoaineallergia gadoliniumille tai sydämen tahdistin. Tarkempi vierasesineiden ja vasta-aiheiden kartoitus tehdään HUS:n ohjeen mukaisesti ennen kuvantamista.

Haju-ja makuaistin tutkimus

Tutkimus tehdään lyhyenä versiona, jossa tehdään hajuaistin seulontatesti, hajukynnyksen testaaminen ja perusmakujen testaus. Testaaminen tehdään tutkimushoitajan toimesta samalla käynnillä, jolloin tehdään MRI-kuvantaminen. Testaamisessa käytetään hajun seulontatestiä ja Sniffin´Sticks (II tason) testin hajukynnyksen testausta sekä makuaistin testaamiseen tarkoitettuja liuskatestejä.
Vasta-aiheet: Tunnettu allergia jollekin käytetylle hajuaineelle ja ennen koronavirusinfektiota diagnosoitu hajuaistin häiriö.

Keuhkofunktio ja suorituskyky

Pyydämme potilailta suostumuksen heistä kerätyn sen kliinisen datan käyttöön, joka heistä rekisteröidään keuhkosairauksien järjestämän jälkikontrollin yhteydessä. Tämä data sisältää tiedot mahdollisista spirometria- ja diffuusiokapasiteettitutkimuksista, keuhkojen kuvantamisesta ja kliinisistä tiedoista, joita on saatu potilasta haastattelemalla.
Kuuden kuukauden jälkipoliklinikkakäyntiä varten potilaille tehdään tavanomainen spirometria (Pt-FV-SpirD) ja diffuusiokapasiteetin mittaus (Pt-Dco-SB1) kliinisen fysiologian laboratoriossa. Poliklinikkakäynnillä tehdään myös 6 minuutin kävelytesti ja happisaturaation mittaus ilman happilisää ennen ja jälkeen kävelytestin.
Vasta-aiheet, 6 minuutin kävelytesti: Vaikea sydänsairaus, rasituksessa ilmaantuva rintakipu tai vaikea hengenahdistus, happisaturaatio huoneilmalla ennen testiä <90%, vaikea tuki-ja liikuntaelimistön sairaus, joka estää kävelyn tai pahenee rasituksessa, kuumeinen infektio paraikaa.

Verinäytteet

Jälkipoliklinikalle tulevilta potilailta otetaan kliinisin indikaatioin seuraavat näytteet, jotka kuuluvat teho-osaston jälkipoliklinikan rutiineihin: PVK, CRP, krea, Na, K, gluc, Hyyttek. Muut verinäytteet otetaan laboratoriossa ensimmäisen poliklinikkakäynnin yhteydessä tai pian sen jälkeen. Plasma ja seeruminäytteistä analysoidaan valkosolupopulaatiot virtaussytometrialla tai massaspektrometrialla ja määritetään sytokiini- ja vasta-ainetasoja. Ensisijaisesti tähän alatutkimukseen otetaan niitä potilaita, jotka ovat aiemmin osallistuneet tutkimukseen ”VASTA-AINEET UUDEN KORONAVIRUSINFEKTION (SARS-CoV-2 / COVID-19) DIAGNOSTIIKASSA JA HOIDOSSA” akuutin koronavirusinfektionsa aikana, mutta myös muita teho-osastolla hoidettuja vaikean taudinkuvan potilaita. Näin voidaan vertailla akuutin vaiheen immunologista profiilia (kerätty aikaisemmin) samoilla potilailla tilanteeseen nyt 6kk sairastumisesta. Tutkimuksessa analysoidaan myös anergiaa ja muita vaikean sairauden seurauksena kehittyviä pitkäkestoisia immuunijärjestelmän muutoksia. Tutkimusnäytteinä otetaan seuraavat näytemäärät ja laadut: 1x10 ml seerumiputki, 1x10 ml EDTA-putki ja 4 x 10 ml hepariiniputki.

Lisäksi otetaan **1x5 ml EDTA-plasmaa** neurofilamentti -määrityksiä varten. Näytteet pakastetaan ja kun aineisto on kerätty, ne lähetetään Göteborgiin Kaj Blennowin ja Henrik Zetterbergin laboratorioon analysoitavaksi.
ApoE-alleelimääritystä varten tarvitaan **1x5ml kokoverinäyte**, joka pakastetaan suoraan odottamaan analyysiä. Tätä näytettä varten on erillinen tiedote ja suostumusmenettely.

Fibroosi- ja inflammaatiomarkkerianalyyseja (MMP-7, -8, -9 ja TIMP-1 ja niitä säätelevät sytokiinit TNFα, IL-6, IL-8, IL-10) varten kerätään **1x10ml hepariiniplasmaa**. Näytteet analysoidaan Tampereen Yliopistossa dosentti Mari Hämäläisen laboratoriossa.
Veren hyytymisominaisuuksien tutkimiseksi (analysoidaan mm tromboosiin liittyviä mikrovesikkeleitä, kalibroitu automoitu trombografia (CAT)) otetaan **3x 2,5ml sitraattiplasmaa,** joka kaksoissentrifugoidaan ja pakastetaan myöhempää analyysia varten. Pyydämme myös tutkittavien suostumusta mahdollisten aikaisemmista verinäytteistä HUSLAB:n tutkimusluvalla anonyymisti ilman kliinisiä tietoja tehtyjen hyytymistutkimusten tulosten käyttämiseksi tutkimusaineistossa ja tulosten yhdistämiseen kliinisiin tietoihin.

Yhteistyökumppanimme ovat kehittäneet nopeaa ja korkean kapasiteetin testiä SARS-CoV2 immuniteetin mittaamiseen ja tutkimiseen (ei-diagnostinen testi). Testin avulla voidaan määrittää automatisoidusti tekoälyn avulla 1-3 immunoglobuliiniluokkaa näytteistä. Testi vastaa osittain normaalia immunofluoresenssimittausta (IFA), jota käytetään rutiinivirusdiagnostiikassa. Testin avulla tarkoituksemme on tutkia potilasnäytteistä, mitä vasta-aineluokkia SARS-CoV-2-viruksen eri proteiineja vastaan ne ilmentävät infektion eri vaiheissa, ja tarkastella, kuinka nämä korreloivat infektion kliiniseen kuvaan. Lisäksi selvitämme, kuinka kauan vasta-aineita on havaittavissa potilaan näytteissä (eri aikapisteissä kerätyt näytteet) ja mahdollisesti muiden koronavirusten kanssa ristireagoivia vasta-aineita. Tutkimuksessa potilaista otetaan seeruminäytteitä 6kk, 12kk ja 24kk sairaalahoidon jälkeen. Lisäksi potilailta pyydetään suostumusta käyttää heistä mahdollisesti aiemmin, n 3kk sairaalahoidosta, otettuja tutkimusnäytteitä myös tähän tarkoitukseen, mikäli näytteitä on otettu jonkin muun tutkimuksen yhteydessä. Kerättäville näytteille tehdään vasta-ainemääritykset myös muilla tekniikoilla (kuten entsyymi-immunologisella (ELISA) sekä mikroneutralisaatiomenetelmällä.[32, 33] Näytteistä selvitetään myös nk. aviditeettia, eli sitä, kuinka voimakkaasti vasta-aineet sitoutuvat viruksen antigeeniin.
Tuloksia voidaan käyttää hyödyksi tuotekehitystyössä ja diagnostiikan kehittämisessä yhteistyössä alan yritysten kanssa. Tarvittaessa potilaisiin voidaan ottaa uudelleen yhteyttä lisänäytteiden keräämiseksi. Tarvittava näytemäärä on **5ml EDTA**-plasmaa.

Erillisellä näytteenottokerralla, jonka ajankohta määrittyy sydämen magneettitutkimuksen perusteella (näytteet tulee ottaa samana päivänä kuin magneettitutkimus tehdään), otetaan seuraavat verinäytteet ja EKG: PVK, krea, sydänlihasvauriota ja vajaatoimintaa kuvastavat merkkiaineet TnI, pro-BNP.

Vasta-ainetutkimuksen osalta on oleellisinta menetelmän testaaminen ja testitulosten vertaaminen muihin menetelmiin. Tehtävät testit eivät ole diagnostisia. Tulokset ovat viitteellisiä.

Tutkittavat saavat kuitenkin halutessaan tietää tutkimuksesta saadut vasta-ainetuloksensa, joista annetaan myös lausunto.

Vasta-aiheet: Ei vasta-aiheita

IV Aikataulu

Eettisen toimikunnan lausuntohakemus tutkimussuunnitelmasta 5-6/2020
Tutkimuslupaprosessi 5-6/2020
Potilaiden identifiointi ja rekrytointi (kirjeet ja lehti-ilmoitukset) alkaa 6/2020
Ensimmäiset puhelinhaastattelut 6/2020
Ensimmäiset 6kk kontrollikäynnit (2 erillistä käyntipäivää, ks yst taulukko)
Laboratoriotutkimusten analyysit 3-6/2021
Sydämen MRI-tutkimukset 4/2021 mennessä
Tulosten analysointi 1-6/2021
Tutkimustulosten raportointi 6-2021-12/2022

V Työnjako tutkimusryhmän jäsenten kesken

Työnjakoa tutkimusaineiston hankkimisessa kuvataan taulukossa 1.
Hankkeessa syntyy osatöitä kolmen jatko-opiskelijan (H.O., S.K. ja R.P.) väitöskirjoja varten, joita he valmistelevat oman työnsä ohella n 25% työajasta. Mukana testaajina neuropsykologisten testien osalta on 1-2 neuropsykologian pro-graduopiskelijaa. J.H. on hankejohtaja ja H.O.:n ja S.K:n väitöskirjan vastuuohjaajana. Neuropsykologian jatko-opiskelija R.P.:n ohjaajina toimivat prof Laura Hokkanen ja J.H.
Neurologian erl Marjaana Tiainen vastaa 3kk puhelinhaastatteluista yhdessä J.H:n, L.H.:n, S.K.:n ja H.O.:n kanssa ja potilaan neurologisen tilanteen indisoimista mahdollisista jatkoselvittelyistä. Juha Martola suunnittelee tutkimukseen soveltuvan kuvantamisprotokollan ja ohjeistaa kuvantamisen logistiikasta vastaavaa hoitajaa Anne Komsia ja vastaa MRI-kuvantamisen tulosten tulkinnasta yhdessä fyysikko Linda Kuuselan kanssa. Sydänkuvantamisesta vastaavat Sari Kivistö ja Miia Holmström yhdessä Valtteri Uusitalon, Suvi Syvärannan ja Satu Vaaran kanssa ja tarvittaessa kardiologiset arviot tekee Tiina Heliö.

Tehohoitolääkärit J.H., P.P., H.O., S.K. tapaavat potilaan jälkipoliklinikalla, käynnin kesto 45-60 min. Lisäksi H.O ja S.K. tehtäviin kuuluu potilaan tehohoitotietojen rekisteröinti, tulosten analysointi ja raportointi. P.K, H-R.K ja J.P vastaavat keuhkosairauksien jälkiseurannan tietojen rekisteröinnistä ja auttavat identifioimaan verrokkihenkilöt jälkiseurantaan kutsutuista potilaista. M.T. on varahankejohtaja. Hankkeen puitteissa voidaan myös suorittaa 1-2 lääketieteen kandidaatin syventävät opinnot, joista LK Inari Taskila on liittymässä tutkimusryhmään 10/2020 ja osallistuu tiedonkeruuseen, hajuaistin testaukseen ja mm 6 minuutin kävelytestien tekemiseen.
Kävelytestien tekemiseen osallistuu fysioterapeutti Linda Ulenius.
Päivi Piirilä lausuu kliiniset keuhkofysiologiset mittaukset.
Neurofilamenttianalyysit tehdään Ruotsissa Göteborgin Yliopiston tutkijoiden Henrik Zetterbergin ja Kaj Blennowin toimesta.
ApoE-analyysit tehdään Dos Liisa Myllykankaan laboratoriossa HUS:ssa.
Yhteistyökumppaneina toimivat myös prof Kai Kaila ja jatko-opiskelija LL Samu Kurki.
Hyytymistutkimusten analysoinnista vastaavat prof Riitta Lassila, dos Lotta Joutsi-Korhonen ja Tuukka Helin HUSLAB:sta ja LT Mirka Sivula ATeK:n tehoklinikasta.

VI Eettiset näkökohdat

Hankkeen ensisijainen päämäärä on tukea potilaita kriittisestä sairaudesta toipumisessa. Tämä on ollut jälkipoliklinikkatoiminnan tavoite jo vuosia, mutta juuri nyt, kun toimintaa kipeimmin tarvitaan, se on logistisista syistä mahdoton järjestää muuten kuin tutkimusprojektina. Siksi projektiin osallistuminen on mahdollista vain, jos potilas antaa siihen tietoon perustuvan suostumuksensa. Kyseessä ovat usein työikäiset potilaat, joiden paluu normaaliin elämään saattaa olla vaikeutunutta sairauden seurauksena. Covid-19:n sairastaminen seurauksineen on somaattisen haastavuutensa lisäksi psyykkisesti kuormittavaa ja jopa leimaavaa. Tehohoidon aikaiset kokemukset voivat pahimmillaan johtaa post-traumaattiseen stressireaktioon, toimintakyvyn heikkenemiseen ja huonontuneeseen elämänlaatuun. Projekti tähtää siihen, että haitat identifioidaan ja potilaat pääsevät keskustelemaan tehohoitoonsa liittyvistä asioista ja nykyisistä oireistaan. Tarvittaessa heidät ohjataan kuntoutukseen ja löydösten perusteella tarvittaessa hoitoon. Erityisesti neurologiset ja neuropsykologiset oireet sekä sydänoireet ovat toipumisen kannalta keskeisen tärkeitä tunnistaa. Tämä on nähdäkseni erittäin tärkeää. Kaikki kerättävä tieto käsitellään luottamuksellisesti ja analysoidaan pseudonymisoituna ilman, että potilaita voidaan missään vaiheessa tunnistaa.
Aivojen magneettikuvantamisessa tai muissa tutkimuksissa ilmenevät kliinisesti merkittävät löydökset saatetaan tutkimuksesta vastaavan henkilön tietoon ja hän järjestää asianmukaisen arvion ja tarvittaessa hoitoon ohjauksen. Sydänkuvantamisen tulosten suhteen konsultoidaan tarvittaessa kardiologia, ja säteilyn käyttöä edellyttävät tutkimukset (sepelvaltimoiden TT) tehdään kardiologin arvioon perustuen.
ApoE-analyysissa voi paljastua kohonnut riski muistisairauteen, mikä voi olla tutkittavalle vaikeasti käsiteltävä asia. Tästä syystä tutkittavalle, joka antaa suostumuksensa tähän osatutkimukseen, annetaan erillinen tiedote tutkimuksesta ja hänellä on oikeus itse valita haluaako hän saada tiedon tutkimustuloksestaan. Tätä kysytään suostumuslomakkeessa, jossa myös ilmaistaan tutkittavan oikeus muuttaa mieltään asian suhteen myöhemmin. Tutkittaville, jotka saavat tiedon lisääntyneestä riskistään, järjestetään mahdollisuus keskustella asiasta neurologin kanssa.

Eettisesti haastavaa mielestäni on, että neuropsykologisen testauksen tulosten tulkinta on hyvin riippuvaista kielitaidosta ja siksi neuropsykologiseen osatutkimukseen voidaan ottaa vain potilaita, joiden asiointikielenä on suomi. Muun kielisten potilaiden osuus Covid-19 tehohoidossa on ollut kuitenkin suuri. Mikäli projektissa havaitaan suomenkielisten osalta, että tutkittavien joukossa on merkittävästi löydöksiä, pyritään järjestämään tutkimus ainakin osittaisena myös halukkaille muun kielisille potilaille kliinisin perustein.
Toinen eettinen haaste on, että logistisesti mahdollinen tutkimusajankohta on vasta 6kk sairastamisesta, jolloin pahimmassa tapauksessa potilas on saattanut oireilla jo pitkään. Covid-taudin tilanne kesällä 2020 on kuitenkin vaikea ennakoida ja on todennäköistä, että pääsy sairaalan tiloihin ja henkilökuntaresurssi ovat vielä rajattuja esimerkiksi 3kk ensimmäisten potilaiden tehohoidosta. Potilas voi onneksi kertoa haittaavista oireistaan jo 3kk puhelun yhteydessä ja saada siten apua jo aikaisemmin.

VII Tutkimuksen merkitys

Hankkeessa tehohoitopotilas kohdataan tehohoidon jälkeen toipilasvaiheessa ja hänen tilannettaan tarkastellaan kokonaisvaltaisesti ja yksilöllisesti. Potilasta askarruttaviin kysymyksiin pyritään vastaamaan ja hänet ohjataan tarvittaessa hoitoon tai kuntoutukseen. Tehohoidosta selviytyminen elossa ei riitä, vaan potilasta tulee tukea myös toipumisprosessissa. Tehohoidon jälkeiset erityisongelmat ovat usein vieraita niille

terveydenhuollon ammattilaisille, jotka potilas kohtaa sairaalasta kotiuduttuaan. Jälkipoliklinikka toimii parhaimmillaan siltana erikoissairaanhoidon ja perusterveydenhuollon välillä. Lisäksi tutkimuksesta saadaan arvokasta uutta tietoa potilaiden sydänterveydestä sairastetun koronavirusinfektion jälkeen. Sydäntutkimuksissa ja hyytymistutkimuksissa saatava tieto saattaa johtaa lääkityksellisiin muutoksiin, joiden tavoitteena on ehkäistä komplikaatioita. Pitkäaikaisseurannassa saatava tieto neurologisesta sairastavuudesta voi jouduttaa potilaiden pääsyä hoidon ja kuntoutuksen piiriin oikea-aikaisesti.

Projektissa toteutuu laaja yhteistyö niin lääketieteen erikoisalojen kuin eri tieteenalojenkin välillä. Hyödynnämme ja vahvistamme myös jo rakennettua yhteistyötä neuropsykologian tutkijoiden ja tehohoidon tutkijoiden kesken. Hankkeeseen sisältyy myös kansainvälistä yhteistyötä ja se tukee akateemisen urakehityksen edistymistä kolmen väitöskirjaprojektin (kaksi tehölääketieteen ja yksi neuropsykologian alaa) osatöinä. Yhteistyönä Helsingin Yliopiston kanssa hankkeen puitteissa ohjataan 1-2 lääketieteen kandidaatin syventäviä opintoja sekä 1-2 neuropsykologian opiskelijan pro-gradu työtä.

Viitteet:

1) Pandharipande P, Girard T, Jackson J, et al.: Long-term cognitive impairment after critical illness. NEJM 2013 369:1306-16

2) Calsavara A, Costa P, Nobre V, et al.: Factors associated with short and long term cognitive changes in patients with sepsis. Scientific Reports 2018 8:4509

3) Herridge MS, Cheung AM, Tansey CM, et al.: One-year outcomes in survivors of the acute respiratory distress syndrome. NEJM 2003; 348:683-93

4) Herridge MS, Tansey CM, Matté A, et al.: Functional disability 5 years after acute respiratory distress syndrome. N Engl J Med. 2011 Apr 7;364:1293-304. doi: 10.1056/NEJMoa101180

5) Mikkelsen ME, Christie JD, Lanken PN, et al.: The adult respiratory distress syndrome cognitive outcomes study: long-term neuropsychological function in survivors of acute lung injury.Am J Respir Crit Care Med. 2012 Jun 15;185(12):1307-15. doi: 10.1164/rccm.201111-2025OC.

6) Asadi-Pooya AA[1], Simani L: Central nervous system manifestations of COVID-19: A systematic review. J Neurol Sci. 2020 Apr 11;413:116832. doi: 10.1016/j.jns.2020.116832.

7) Mao L, Jin H, Wang M, et al: Neurologic manifestations of hospitalized patients with coronavirus disease 2019 in Wuhan, China. JAMA Neurol 2020 doi: 10.1001/jamaneurol.2020.1127

8) Carod-Artal FJ: Neurological complications of coronavirus and COVID-19. Rev Neurol. 2020 May 1;70(9):311-322. doi: 10.33588/rn.7009.2020179.

9) Helms J, Kremer S, Merdji H, et al.: Neurologic features in severe SARS-CoV2- infection. NEJM 2020, DOI: 10.1056/NEJMc2008597

10) Wihersaari L, Ashton NJ, Reinikainen M, Jakkula P, Pettilä V, Hästbacka J, et al.: Neurofilament light as an outcome predictor after cardiac arrest: a post hoc analysis of the COMACARE trial. Intensive Care Med 2020: https://doi.org/10.1007/s00134-020-06218-9

11) Khalil M, Teunissen CE, Otto M, Piehl F, Sormani MP, Gattringer T, et al. : Neurofilaments as biomarkers in neurological disorders Nat Rev Neurol. 2018 Oct;14(10):577-589.

12) Kanberg N, Ashton NJ, Andersson LM, Yilmaz A, Lindh M, Nilsson S, et al.: Neurochemical evidence of astrocytic and neuronal injury commonly found in COVID-19. **N**eurology. 2020 Jun 16:10.1212/WNL.0000000000010111. doi: 10.1212/WNL.0000000000010111.

13) Kuo C-L, Pilling LC, Atkins JL, Kuchel GA, Melzer D. ApoE e2 and aging-related outcomes in 379,000 UK Biobank participants. medRxiv [Internet]. 2020. http://medrxiv.org/content/early/2020/02/13/2020.02.12. 20022459.abstract. Accessed May 4, 2020

14) Kuo C-L, Pilling L, Atkins JL , Masoli JAH, Delgado J, Kuchel GA, et al.: APOE e4 Genotype Predicts Severe COVID-19 in the UK Biobank Community Cohort. J Gerontol A Biol Sci Med Sci, 2020, Vol. XX, No. XX, 1–2 doi:10.1093/gerona/glaa131

15) Vrsalovic M, Vrsalovic Presecki A. Cardiac troponins predict mortality in patients with COVID-19: A meta-analysis of adjusted risk estimates. J Infect. 2020;S0163-4453(20)30300-5.

16) Petrilli CM, Jones SA, Yang J, et al. Factors associated with hospital admission and critical illness among 5279 people with coronavirus disease 2019 in New York City: prospective cohort study. BMJ. 2020;369:m1966.

17) Siripanthong B, Nazarian S, Muser D, Deo R, Santangeli P, Khanji MY, Cooper LT Jr, Chahal CAA. Recognizing COVID-19-related myocarditis: the possible pathophysiology and proposed guideline for diagnosis and management. Heart Rhythm. 2020 May 5.

18) Szekely Y, Lichter Y, Taieb P, et al. The Spectrum of Cardiac Manifestations in Coronavirus Disease 2019 (COVID-19) - a Systematic Echocardiographic Study [published online ahead of print, 2020 May 29]. Circulation. 2020

19) Tang N, Bai H, Chen X, ym. Anticoagulant treatment is associated with decreased mortality in severe coronavirus disease 2019 patients with coagulopathy. J Thromb Haemost 2020;18:1094¤#x2013;9.

20) Zhang L, Yan X, Fan Q, ym. D-dimer levels on admission to predict in-hospital mortality in patients with Covid-19. J Thromb Haemost, julkaistu verkossa 19.4.2020. DOI:10.1111/jth.14859.

21) Whitcroft KL, Hummel T. Olfactory Dysfunction in COVID-19. Diagnosis and Management. JAMA. Published online May 20, 2020. doi:10.1001/jama.2020.8391

22) Philpott C, DeVere D. 2014. Postinfectious and post-traumatic olfactory disorders. In: Welge-Luessen A, Hummel T, editors. Management of smell and taste disorders. New York: Thieme; p. 91–105.

23) Sorokowska A, Drechsler E, Karwowski M, Hummel T. Effects of olfactory training: a meta-analysis. Rhinology 2017 Mar 1;55(1):17-26.

24) Rombaux P, Huart C, Deggouj N, Duprez T, Hummel T. Prognostic Value of Olfactory Bulb Volume Measurement for Recovery in Postinfectious and Posttraumatic Olfactory Loss. Otolaryngol Head Neck Surg 2012 Dec;147(6):1136-41.

25) Ueland T, Holter JC, Holten AR, Müller KE, Lind A, Bekken GK, et al.: Distinct and early increase in circulating MMP-9 in COVID-19 patients with respiratory failure. J Infect 2020; 81:e41-e43

26) Lauhio A, Konttinen YT, Tschesche H, Nordström D, Salo T, Lähdevirta J, et al.:Reduction of matrix metalloproteinase 8-neutrophil collagenase levels during long-term doxycycline treatment of reactive arthritis. Antimicrob Agents Chemother 1994; 38: 400-2

27) Sivula M, Hästbacka J, Kuitunen A, Lassila R, Tervahartiala T, Sorsa T, et al.: Systemic matrix metalloproteinase-8 and tissue inhibitor of metalloproteinases-1 levels in severe sepsis-associated coagulopathy. Acta Anaesthesiol Scand 2015;59: 176-84

28) Hästbacka J, Linko R, Tervahartiala T, Varpula T, Hovilehto S, Parviainen I, et al.: Serum MMP-8 and TIMP-1 in critically ill patients with acute respiratory failure: TIMP-1 is associated with increased 90-day mortality.
Anesth Analg. 2014;118:790-8.

29) Schuster A, Hor KN, Kowallick JT, Beerbaum P, Kutty S. Cardiovascular Magnetic Resonance Myocardial Feature Tracking: Concepts and Clinical Applications. Circ Cardiovasc Imaging. 2016;9:e004077.

30) Patel AR, Kramer CM. Role of Cardiac Magnetic Resonance in the Diagnosis and Prognosis of Nonischemic Cardiomyopathy. JACC Cardiovasc Imaging. 2017;10:1180-1193.

31) Toubiana, J. et al. Outbreak of Kawasaki disease in children during COVID-19 pandemic: a prospective observational study in Paris, France. Preprint at medRxiv https://doi.org/10.1101/2020.05.10.20097394 (2020)

32) Amanat F, Stadlbauer D, Strohmeier S, Nguyen T, Chromikova V, McMahon M, et al.: A serological assay to detect SARS-CoV-2 seroconversion in humans. Nat Med 2020; 26:1033-36

33) Haveri A, Smura T, Kuivanen S, Österliund P, Hepojoki J, Ikonen N, et al.: Serological and molecular findings during SARS-CoV-2 infection: the first case study in Finland, January to February 2020 . Euro Surveill 2020; 25(11):2000266. doi: 10.2807/1560-7917.ES.2020.25.11.2000266
