# Supplementary material for: Cardiac magnetic resonance -detected myocardial injury is not associated with long-term symptoms in patients hospitalized due to COVID-19
Source: PLoS One. 2023 Mar 8;18(3):e0282394. doi: 10.1371/journal.pone.0282394 (PMC9994679; doi:10.1371/journal.pone.0282394)
Supplement: S2 Protocol — (DOCX) [file pone.0282394.s003.docx]

**Research Protocol: Recovery from critical coronavirus infection**

**I Background**

Long-term survival after intensive care has risen to an important position in the field's research in recent years. It takes a long time for a patient to recover from a critical illness, and the risk of death for those who leave the hospital alive is elevated for a long time. In addition, patients can be left with many symptoms that impair the quality of life from a critical illness, its treatment methods, and an extended stay in the hospital. After intensive care, deterioration of cognitive function corresponding to moderate Alzheimer's disease has been described even in young and previously healthy individuals^1^.

Not all the reasons for this are known, but for example, quite common (up to 80% ) confusion or delirium during intensive care has been identified as a risk factor^1,2^ In our ongoing research project in ASSESS- shock 2 (TYH2020341), we are investigating the connection of circulatory failure with delirium and neuropsychological recovery, as well as changes in the body's microbiome during intensive care. The COVID-19 caused by the coronavirus has also caused a significant increase in the need for intensive care among working-age people. There is still no information on the adverse effects of long-term follow-up about COVID-19 pneumonitis. Still, severe respiratory failure (ARDS, acute respiratory distress syndrome) is known to occur in the long-term as shortness of breath during exertion and deterioration of cognitive performance even one or several years after hospitalization ^3,4,5^. Based on our own clinical experience and the literature accumulated with COVID-19 patients, in addition to severe respiratory failure, a considerable number of neurological complications occur in COVID-19 patients^6,7^. Delirium is prevalent and long-lasting; it is probably related to heavy long-term ventilatory therapy and the long-term use of anesthetics and opioids required. It is also possible that the coronavirus affects the central nervous system directly and increases the risk of several other neurological complications, such as convulsions, encephalitis, cerebral circulation disorders, and polyradiculitis^8,9^.

New laboratory markers are being used in research to assess brain damage. Neurofilament (light) (neurofilament light, NFL) is an intra - axonal protein that leaks out of degenerating axons. This increases the NFL concentration in the cerebrospinal fluid and blood in patients with central nervous system disease involving neuronal damage. Cerebrospinal fluid and blood concentrations correlate well with each other in different neurological disease states and with the severity of the disease. The latter has been demonstrated in neurodegenerative diseases, but also recently in acute hypoxia in ischemic damage in cardiac arrest patients^10^. The neurofilament concentration increases slowly within weeks and remains abnormal for months. NFL is currently considered the best marker of neurodegeneration because it has few confounding sources of error^10, 11^. Plasma NFL has been found to rise in connection with acute neurological damage and in the acute phase of Covid-19^12^.

ApoE lipoprotein is a lipoprotein involved in the body's fat metabolism, which is also linked to the risk of hypertension and atherosclerosis and the risk of delirium and neurodegenerative diseases such as Alzheimer's disease^13^. It has six allelic types, ApoE2, ApoE3, and ApoE4, which occur in six combinations. In the European population, about 15-20 % of the population has the ApoE4 allele, and homozygotes have a 14-fold risk of developing Alzheimer's disease. ApoE4 has been shown in vitro to be associated with easier entry of certain virus types into the cell. In a recently published study based on biobank data, it was concluded that the ApoE4 allele seemed to predispose patients to a more severe disease picture of COVID-19 when the underlying diseases associated with it were considered in the analysis^14^. The connection to neurological complications in the acute phase of COVID-19 or as late complications after a critical illness is not known. To our knowledge, there are no previous studies on the subject.

Cardiac enzymes have been reported to be elevated in approximately 12-35% of hospitalized COVID-19-patients, especially in severe disease, presumably based on infection-related myocarditis (myocarditis), ventricular overload, or ischemia^15,16^. Myocarditis can be asymptomatic or present as arrhythmias, progressive failure, and cardiogenic shock^17^. So far, there is no information on the long-term effects of COVID-19-infection on cardiovascular health, such as post-infection scars and myocardial function. Also, severe pulmonary dysfunction and the risk of pulmonary embolism can burden the right ventricle^18^. The prothrombotic tendency is increased in patients with COVID-19. Already in the early stages of the pandemic, the activation of the coagulation system was found to have a connection with mortality. In the acute phase of the disease, high D-dimer is strongly connected to an increased risk of death^19,20^. As far as we know, there is still no comprehensive research data on the recovery of the COVID-19 patient’s coagulation system in longer-term follow-up.

For ten years now, our intensive care outpatient clinic has monitored the recovery of patients, especially after long periods of intensive care, and reviewed their experiences during intensive care with patients. Based on our unpublished data, 2-3 months after intensive care, patients report still suffering from many symptoms affecting their quality of life, such as insomnia, nightmares, depression, apathy, exhaustion, and cognitive problems. Memories of delirium are common and disturbing, and going through them with patients is crucially important, and just saying them and declaring them normal helps many. Currently, the intensive care outpatient clinic is temporarily on hiatus due to the challenges brought by the information system change. COVID patients risk missing out on an essential follow-up appointment with an intensive care physician. For this reason, we now want to organize a follow-up outpatient clinic visit as a research project, where we go over issues related to intensive care and his current condition with the patient and, at the same time, investigate neurological and neuropsychological recovery related to long-term recovery from intensive care with clinical tests and imaging methods. The outpatient clinic visit also examines the healing of physical performance, the respiratory system, and immune defenses from a severe illness. It investigates heart symptoms that appeared during or after the disease.

Smells evoke strong positive and negative feelings. Thus, the loss of the sense of smell can significantly worsen the quality of life. Upper respiratory tract infection caused by a virus is the most common cause of loss of sense of smell. During the COVID-19 pandemic, changes in the sense of smell and taste have even been a primary symptom in some of those affected by the coronavirus infection (SARS-CoV-2)^21^. 32-66 % of olfactory disorders after an upper respiratory tract infection recover spontaneously^22^. Olfactory training has been shown to help the sense of smell in recovery^23^. The size of the olfactory coil has been shown to have a predictive value regarding the recovery of the sense of smell^24^. In some patients with coronavirus infection, the sense of smell has recovered within two weeks. Long-term follow-up regarding the recovery of the sense of smell is currently unknown. The study also evaluates whether the continuation of the hyperinflammatory state can be related to prolonged symptoms after COVID-19.
Matrix metalloproteinases (MMP) -7 and -9 have been described to occur in elevated concentrations in many chronic lung diseases, and recently MMP-9 was also shown to be associated with more severe COVID-19 respiratory failure^25^. MMPs are involved in modifying the intercellular space, e.g., of neutrophils endopeptidases released from granulocytes, which have multiple roles in acute and chronic inflammation. MMP-8 levels, among others, are associated with reactive arthritis and coagulation disorders^26, 27^. MMP regulation involves, among other things, proinflammatory cytokines, and at the tissue level, they are regulated by MMP tissue inhibitors, TIMPs. TIMP-1 has been described as an independent prognostic factor with 1-year mortality in patients with acute respiratory failure^28^.

**II Research questions, hypothesis, research goal**

The study aims to find out (Q=the question layout, H=hypothesis):

1) Q: Do patients treated in the intensive care unit due to the coronavirus show a decline in cognitive performance revealed in extensive neuropsychological tests compared to reference values determined from the general population?

H: Neuropsychological tests show changes in some patients compared to the general population of the same age group.

2) Q: Did any clinically observed neurological complications occur during intensive care?

H: Neurological complications such as delirium and cerebrovascular disorders occurred during intensive care.

3) Q: How have patients recovered from a severe illness neurologically?

H: Most of them have recovered well neurologically, but some may find symptoms in a detailed examination.

4) Q: Does the MRI examination show changes compared to the COVID-19 patients who survived without intensive care treated in the ward?

H: Patients with neurological abnormalities on clinical examination may have radiologically detectable changes on head MRI.

5) Q: Are the patients' neurological symptoms related to white matter damage that could be detected by determining neurofilament levels?

H: Patients with neurological or neurocognitive symptoms can be detected in plasma neurofilament levels compared to non-COVID patients and COVID patients who are neurologically asymptomatic.

6) Q: Is the ApoE4 allele more common in patients with severe Covid-19?

H: In intensive care and hospitalized Covid-19 patients, one or two ApoE4 alleles are detected more often than in patients with Covid-19 at home or controls who have not had a coronavirus infection.

7) Q: Do elevated neurofilament levels and any ApoE allele predict long-term neurological symptoms?

H: Patients with an elevated neurofilament level 6 months after the disease have more neuropsychological changes five years after the infection.

8) Q: Do the patients have a weakening sense of smell or taste compared to the average population 6sixmonths after becoming ill and are there any changes in the neural pathways of the sense of smell on MRI?

H: Some patients have changes in their sense of smell and taste, which have not recovered well or only partially after six months of illness. An MRI examination shows changes in the olfactory nerve or neural pathway.

9) Q: What are the patients' physical performance and lung function parameters 3 and 6 months, and two years after intensive care?

H: In some patients, lung function and physical performance have not recovered to the level of reference values calculated from an average population of the same age.

10) Q: What is the state of the patient’s immune system (is there long-term anergy or other leukocyte dysfunction six months after intensive care)?

H: The functioning of the immune system may still be weakened after six months of illness.

11) Q: Do those iin intensive careor inpatient unit have cardiovascular symptoms?

H: During and/or after the coronavirus infection, there are cardiac symptoms, and they have been more common and continued longer in patients treated in intensive care in demanding patients.

12) Q: Are myocarditis changes visible on magnetic resonance imaging of the heart, and are they present in connection with patients' poorer recovery and cardiac symptoms?

H: Fatigue and slow performance in some patients, especially those who needed intensive care, the background of recovery may be detectable by magnetic resonance imaging sequelae of myocarditis.

13) Q: Can changes in blood clotting factors be seen in Covid-19 infection in sick patients six months after the acute illness phase compared to clinical coagulation factor samples taken in the acute phase?

H: Blood coagulation factors have normalized six months after becoming ill.

14) the partial study investigating SARS-CoV-2 immunity aims to develop tests to measure COVID-19 immunity and bring additional information about the infection immunity and its duration. This is also important for the Covid-19 vaccine in monitoring effectiveness, as the test can be used to measure the vaccine separately induced and infection-induced immune response.

15) Q: Can elevated MMP -7, -8, or-9 concentrations or cytokines or tissue inhibitors involved in their regulation have abnormal concentrations, and whether the potentially elevated levels are associated with clinical symptoms and findings, e.g., lung function or imaging?

H: In some patients who are still severely symptomatic, there are elevated levels of inflammatory markers. The goal is to characterize the patients' long-term recovery and, if necessary, refer the patients to appropriate follow-up examinations or rehabilitation.

**III Research plan, methods, and implementation**

The study population is adult patients treated for coronavirus infection in intensive care units of the HUS area who meet the inclusion criteria and have no contraindications to the study.

**Admission criteria:**

- 18 years old

- the language of business is Finnish or Swedish. For the neuropsychological part of the study, only persons whose business language is Finnish can be included because there are no validated versions of tests for Finnish-Swedes and the test results are very language sensitive. In all other sub-investigations, persons whose business language is Swedish can also be included.

-is in intensive care in one of HUS's intensive care units due to a severe coronavirus infection

-Is involved in the RECOVID study

**Exclusion criteria:**

-pregnancy

- a significant neurological disease diagnosed before the coronavirus infection, such as a severe one

the aftermath of a brain injury or cerebrovascular accident

-sensory impairment

- memory disorder

-Parkinson's disease

-disability

Section-specific exclusion criteria, if any, are listed in connection with the description of each study section. The assumed number of patients is around 100-150. Patients are identified after intensive care using a registered COVID-19 diagnosis from the intensive care quality database, after which they are contacted and asked to participate in the study. Patients are sent a *Patient and Next of kin Research Information Sheet* and a consent form with the contact information of the research team for further details. For the continuation of the study, new information about the subject and consent forms will be sent to the subjects by post, or their consent will be asked when meeting them at the follow-up clinic. Among the research subjects, there are only people who use Finnish as their business language, so the research documents for further research have only been prepared in Finnish.

Control patients are recruited through a newspaper advertisement and with the help of social media. Control patients are persons with a confirmed coronavirus infection whose severity of illness did not require intensive care, but the disease was contracted at home (N=50). Another control group, patients who received treatment for coronavirus infection in a hospital bed ward (N=50), will be identified to ask for consent from those who receive/are receiving an invitation to a follow-up organized by the lung disease clinic. Separate written consent to participate in the study is requested from the control persons.

We also recruited 50 control subjects who had not had a COVID-19 infection. The table below shows the sub-studies of the study in which these so-called non-Covid controls will be compared to other subjects. The aim is to find non-Covid controls by September 14, 2020, from the close circle of the subjects who gave their consent to the study so that they would be as similar as possible to the subjects treated in the intensive care unit in terms of socioeconomic status and educational level. In practice, the subjects are asked at the follow-up clinic if there are people in their close circle who have not had COVID-19 and whom they would estimate would be willing to participate in the study. They will be forwarded the contact information of the research team so that they can express their interest in the research if they wish. After this, possible non-COVID-19 control subjects will be sent the study information and a consent form in a return envelope.

If enough non-COVID controls are not found through this method, they can also be found among the staff of the University of Helsinki from different professional groups by announcing the recruitment on the University's intranet pages. The aim is to obtain controls from different age and professional groups so that the educational level does not differ significantly from the subjects (who were in intensive care due to Covid).

Patients who give their consent will receive an invitation to come for examination and clinic visits six months after intensive care. Before visiting the outpatient clinic, three months after the disease, the research patients are given a short neurological, and a neuropsychological survey by phone and two questionnaires are mailed to them. More extensive inquiries are made at two separate visits six months after intensive care. The attached table shows the time points and the maps planned for them. At Uppsala Academic Hospital and Oslo University Hospital (if possible), a part of the table will be performed for the corresponding patient groups for the mentioned tests, and the data for them will be combined. mRS, RAND-36, MOCA, and IQCODE tests are conducted in Uppsala. Control patients for the MRI study are searched for using a newspaper advertisement, after which, based on contact, they are asked for informed consent to participate in the study.

Table 1. Tests used in the study, division of labor, time points, and subjects number in HUS. *=Separate consent

| Helsinki |  |  |  |
| --- | --- | --- | --- |
| Three months | Who makes | Number of patients | Controls |
| Registration of clinical data from patient records (neurological symptoms and examinations during intensive care, delirium, duration of treatment and procedures, background information) | Johanna Hästbacka Henriikka Ollila Salla Kattainen Minna Tallgren Erika Wilkman | 100-150 | 50 who were in ward treatment |
| Telephone interview: modified Rankin Scale (mRS) | Marjaana Tiainen Laura Hokkanen Sanna Koskinen Johanna Hästbacka Henriikka Ollila | 100-150 | 50 inpatients + 50 COVID patients sick at home |
| Telephone interview: Montreal Cognitive Assessment (T-MoCA) | -“- | 100-150 | 50 inpatients + 50 COVID patients who got sick at home + 50 non-COVID controls |
| Behavior Rating Inventory of Executive Function, adult version (BRIEF-A) +BRIEF Informant version ABNAS Neuropsychological questionnaire | by post | 100-150 | ,, |
| Six months | by post |  |  |
| RAND-36 | by post | 100-150 | 50 inpatients + 50 COVID patients sick at home 50 + non-COVID controls |
| MFI-20 | by post | 100-150 | ,, |
| HADS | by post | 100-150 | ,, |
| Informant Questionnaire on Cognitive Decline (IQCODE-CA) | by post | 100-150 | 50 inpatients + 50 COVID patients who got sick at home |
| Behavior Rating Inventory of Executive Function, adult version (BRIEF-A) +BRIEF Informant version | By mail (or NPS in connection with the study) | 100-150 | 50 inpatients + 50 COVID patients sick at home + 50 non-COVID controls |
| Background information | visit (see Visit 1 below) | 100-150 | ,, |
| modified Rankin Scale ( mRS) | visit | 100-150 | ,, |
| Montreal Cognitive Assessment ( MoCA ) | visit | 100-150 | ,, |
| National Institute of Health Stroke Score (NIHSS) | visit | 100-150 | ,, |
| CFS-9 ( clinical frailty scale ) | visit | 100-150 | ,, |
| Olfactory screening test | visit | 100-150 | ,, |
| Follow-up of lung diseases (with the patient's consent, data retrospectively for comparison) | Lung x-ray, spirometry, and diffusion capacity measurement, telephone interview (skin and allergy hospital) | 100-150 | Data from clinical lung function tests of 50 patients who were in ward treatment |
| **Visit 1** |  |  |  |
| Neuropsychological research Haartmaninkatu 3 | Student /Laura Hokkanen/Sanna Koskinen/ Riikka Pihlaja/ Annamari Tuulio -Henriksson | 100-150 | 50 inpatients + 50 COVID patients sick at home + 50 non-COVID controls |
| Laboratory samples (immunology) | HUSLAB | 10-50 |  |
| Brain MRI (ULS) | HUS imaging Anne Komsi, Juha Martola , Linda Kuusela | 50 who were in intensive care (at least 30) | 50 inpatients + 50 COVID patients sick at home + 50 non-COVID controls |
| Smell and taste testing | Research nurse | 50 who were in intensive care | -”- |
| **Visit 2** |  |  |  |
| 6-minute walk test | Research nurse, physiotherapist Linda Ulenius , ERL Salla Kattainen , LK Inari Taskila (6mwt) | 100-150 |  |
| Spirometry and diffusion capacity, meeting with the intensive care physician, reporting the results, and discussing possible further plans | Päivi Piirilä, LK Inari, Johanna Hästbacka, / Salla Kattanen / Henriikka Ollilla | 100-150 |  |
| Laboratory samples Fibrosis/inflammation markers Neurofilament + ApoE alleles* | MMP and TIMP-1 analyzes Mari Hämäläinen TAU Kaj Blennow , Henrik Zetterberg (University of Gothenburg) Liisa Myllykangas | 100-150 | 50 inpatients + 50 COVID patients sick at home + 50 non-COVID controls |
| Method testing of antibody assays Hyyttek , CAT= calibrated automated thermogram , microvesicles, PF4 | Vilja Pietiäinen Jussi Hepo-oja Olli Vapalahti Riitta Lassila Mirka Sivula Lotta Joutsi-Korhonen (HUSLAB) Tuukka Helin (HUSLAB) | 100-150 | 50 inpatients + 50 Covid patients sick at home + 50 non-COVID controls |
| 6-12 months |  |  |  |
| Magnetic resonance imaging of the heart | HUS imaging Valtteri Uusitalo, Suvi Syväranta, Satu Vaara, Sari Kivistö, Miia Holmström | 50 who were in intensive care, 50 who were in bed ward treatment | 50 non-COVID controls |
| Heart examination laboratory samples P- TnI, Nt -BNP, total blood count, creatinine, ECG | HUSLAB | 50 who were in intensive care, 50 who were in bed ward treatment | 50 non-COVID controls |
| 12 months |  |  |  |
| Antibody assay follow-up samples |  | 100-150 | 50 inpatients + 50 COVID patients who got sick at home |
| 24 months |  |  |  |
| Antibody assay follow-up samples |  | 100-150 | 50 inpatients + 50 COVID patients who got sick at home |
| 6 MW | physiotherapist Linda Ulenius | 100-150 intensive care patients |  |
| Five years |  |  |  |
| Outpatient visits and neuropsychological testing | Intensive care clinic researchers Johanna Hästbacka, TBA | 100-150 | 50 inpatients + 50 COVID patients sick at home 50 non-COVID controls |

*Imaging*

A neuroradiologist evaluates brain magnetic resonance images for visible structural changes(e.g., infarcts and hemorrhages). Neuroradiologist Juha Martola designs an imaging protocol suitable for the study with physicist Linda Kuusela and instructs the nurse responsible for imaging logistics, Anne Koms, and is responsible for interpreting the results of MRI imaging. In addition, the physicist Linda Kuusela further processes the image material in Meilahti's physicists' image laboratory (e.g., Slicer and Freesurfer), where the aim is to illustrate, e.g., damage to the olfactory nerve, damage to nerve pathways and gray/white matter atrophy. To compare the imaging findings with the results obtained in neuropsychological tests, at least 30 patients participating in MRI imaging must also be included in the neuropsychological part of the study, i.e., their language of communication should be Finnish. If coincidental findings, such as cerebral artery aneurysm or other potentially clinically important findings, are found in the magnetic resonance imaging of the brain, Juha Martola will contact the person in charge of the study, who, together with neurologist Marjaana Tiainen, will arrange the necessary follow-up investigations and a referral to adequate treatment. Contraindications: Severe narrow space scar, heart pacemaker, metallic foreign objects in the body, or other known contraindications for MRI examination. More detailed mapping of contraindications is done according to the HUS guidelines before imaging.

Magnetic resonance imaging of the heart takes place 6-12 months after the end of hospital treatment. Cardiac radiologists Sari Kivistö and Miia Holmström are responsible for cardiac magnetic resonance imaging, with specialists Valtteri Uusitalo, Suvi Syväranna, and Satu Vaara, who specialize in cardiac radiology. The imaging is performed by the clinical myocarditis imaging protocol of the radiology heart imaging unit HUS Imaging with a 1.5 Tesla device. The volumes of both chambers and the ejection fraction are measured based on the volumetry of the short-axis cine images. In addition, local myocardial deformation is measured from longitudinal and short-axis images using strain methods^29^. Myocardial edema and fibrosis are measured quantitatively from T1- and T2-weighted relaxation time maps^30^. The localization and percentage of the possible left ventricular scar are determined from gadolinium-enhanced images. Subjects with troponin release during ward treatment or a myocardial scar characteristic for a previously undiagnosed myocardial infarction will be offered computed tomography (CT) of the coronary arteries at the clinical discretion of the cardiologist. However, clinical suggestions are not made if the coronary condition is known from previous examinations. Coronary arteries are assessed for the number of hemodynamically significant coronary plaques, the extensiveness of atherosclerosis, and possible coronary aneurysms, such as having been associated with coronavirus infection in children and adolescents.31 If changes are found, docent, cardiology specialist Tiina Heliö programs the necessary follow-up examinations.

Exclusion criteria for cardiac magnetic resonance imaging: Permanent cardiac pacemaker or severe ischemic or other heart muscle disease already diagnosed before the illness. Contraindications: Severe claustrophobia, allergy to gadolinium or heart pacemaker. More detailed mapping of possible ferromagnetic implants and contraindications is done according to the HUS instructions before imaging each study patient.

*Smell and taste*

The examination is done in a short version, where a screening test for the sense of smell, testing the smell threshold and testing basic tastes. The research nurse does the testing at the same visit when the MRI imaging is done. The testing uses the smell screening test and the smell threshold testing of the Sniffin'Sticks (level II) test, as well as strip tests for the sense of taste. Contraindications: Known allergy to one of the odorants used and a disorder of the sense of smell diagnosed before the coronavirus infection.

*Pulmonary function and performance*

We ask the patients for consent to use the previous clinical data collected. This data includes possible spirometry and diffusing capacity studies, lung imaging, and clinical information obtained by interviewing the patient. The patients undergo standard spirometry (Pt-FV- SpirD) and diffusion capacity measurement (Pt-Dco-SB1) for the six-month follow-up outpatient clinic visit in the clinical physiology laboratory. At the outpatient clinic visit, a 6-minute walk test and measurement of oxygen saturation without supplemental oxygen are also performed before and after the walk test. Contraindications, 6-minute walk test: Severe heart disease, chest pain or severe shortness of breath during exertion, oxygen saturation in room air before the test < 90%, severe musculoskeletal disease that prevents walking or worsens during work, and febrile infection.

*Blood samples*

The following samples are taken from patients who come to the outpatient clinic with clinical indications, which are part of the routines of the outpatient clinic in the intensive care unit: total blood count, CRP, creatinine, natrium, kalium, glucose, and coagulation studies. Other blood samples are taken in the laboratory during the first clinic visit or soon after. Flow cytometry or mass spectrometry analyzes plasma and serum samples for white blood cell populations, and cytokine and antibody levels are determined. Primarily, this sub-study includes patients who have previously participated in the study "ANTIBODIES IN THE DIAGNOSTIC AND TREATMENT OF THE NEW CORONAVIRUS INFECTION (SARS-CoV-2 / COVID-19)" during their acute coronavirus infection, but also other patients with a severe disease treated in the intensive care unit. In this way, the immunological profile of the acute phase (collected earlier) in the same patients can be compared to the situation now six months after becoming ill. The research also analyzes anergy and other long-term changes in the immune system that develop as a result of severe illness. The following sample quantities and qualities are taken as research samples: 1x10 ml serum tube, 1x10 ml EDTA tube, and 4 x 10 ml heparin tube. In addition, 1x5 ml of EDTA plasma is taken for neurofilament determinations. The samples are frozen, and when the material is collected, they are sent to Kaj Blennow and Henrik Zetterberg's laboratory in Gothenburg for analysis. For the ApoE allele determination, a 1x5ml whole blood sample is required, frozen directly to await examination. There is a separate notice and consent procedure for this sample. For fibrosis and inflammation marker analyzes (MMP-7, -8, -9, and TIMP-1 and their regulating cytokines TNFα, IL-6, IL-8, IL-10), 1x10ml of heparin plasma is collected. The samples are analyzed at the University of Tampere in the laboratory of docent Mari Hämäläinen. To study blood coagulation properties(e.g., microvesicles related to thrombosis are analyzed, calibrated automated thrombography (CAT)), 3x 2.5ml citrate plasma is taken, which is double centrifuged and frozen for later analysis. We also ask for the subjects' consent to use the results of coagulation studies performed anonymously without clinical data from previous blood samples with HUSLAB's research permit in the research material and to combine the results with clinical data. Our partners have developed a fast and high-capacity test for measuring and studying SARS-CoV2 immunity (non-diagnostic test). The test can be used to determine 1-3 automatically with the help of artificial intelligence immunoglobulin class from the samples. The test partially corresponds to the standard immunofluorescence assay (IFA) used in routine viral diagnostics. With the help of the test, our purpose is to study which classes of antibodies against different proteins of the SARS-CoV-2 virus they express in patient samples at various stages of the infection and to examine how these correlate with the clinical picture of the disease. In addition, we find out how long antibodies are detectable in the patient's samples (samples collected at different time points) and possibly antibodies that cross-react with other coronaviruses. In the study, serum samples are taken from the patients six months, 12 months, and 24 months after hospitalization. In addition, the patients are asked for their consent to use any research samples taken from them previously, about three months after hospitalization, and also for this purpose, if the samples were taken in connection with another study. The collected samples are also tested for antibodies using other techniques (such as enzyme immunoassay (ELISA) and the microneutralization method.32, 33 The so-called avidity of the samples is also determined, i.e., how strongly the antibodies bind to the virus antigen.

The results can be used to advantage in product development work and diagnostics development in cooperation with companies in the field. If necessary, patients can be contacted again to collect additional samples. The required sample amount is 5 ml of EDTA plasma. At a separate sampling session, the time of which is determined based on the cardiac magnetic examination (the samples must be taken on the same day as the magnetic examination), the following blood samples and ECG are taken: total blood count, creatinine, biomarkers reflecting heart muscle damage and dysfunction hs-TnI and pro-BNP. Regarding antibody studies, the essential thing is to test the method and compare the test results with other methods. The tests performed are not diagnostic. The results are indicative. However, if they wish, the test subjects can know their antibody results obtained from the test, and a statement will also be given about them.

Contraindications: No contraindications

**IV Schedule**

Application for the opinion of the Ethics Committee on the research plan 5-6 /2020

Research permit process 5-6 /2020

Patient identification and recruitment (letters and newspaper announcements) will start on 6/2020

First phone interviews 6/2020

The first 6-month control visits (2 separate visit days, table 1)

Analyzes of laboratory studies 3-6 /2021

Cardiac MRI examinations by 4/2021

Analysis of results 1-6 /2021

Reporting of research results 6-2021 -12/2022

**V Division of work among the members of the research group**

The division of labor in acquiring research material is described in table 1. In the project, part-time work is created for the dissertations of three graduate students), which they prepare in addition to their work for approximately 25% of the working time. 1-2 neuropsychology graduate students do the neuropsychological tests. JH is the project leader and supervisor of HO and SK's dissertation. Neuropsychology graduate students RP’s supervisors are prof Laura Hokkanen and JH. Neurologist Marjaana Tiainen is responsible for the 3-month telephone interviews with JH, LH, SK, and HO and possible follow-up investigations indicated by the patient's neurological situation. Juha Martola designs an imaging protocol suitable for research and instructs Anne Koms, the nurse responsible for imaging logistics, and is responsible for interpreting MRI imaging results with physicist Linda Kuusela. Sari Kivistö and Miia Holmström, together with Valtteri Uusitalo, Suvi Syväranta, and Satu Vaara, are responsible for cardiac imaging and, if necessary, Tiina Heliö makes cardiological assessments for additional treatments.

Intensive care doctors JH, PP, HO, and Sand K meet the patient at the outpatient clinic; the duration of the visit is 45-60 min. In addition, the tasks of HO and SK include registering the patient's intensive care data and analyzing and reporting the results. PK, H – RK, and JP are responsible for registering lung disease follow-up data and helping to identify control subjects from the patients invited for follow-up. MT is the deputy project manager. In the framework of the project, 1-2 medical bachelor's advanced studies can also be completed, of which LK Inari Taskila is joining the research group in 10/2020 and will participate in data collection, olfactory testing, and, for example, 6-minute walking tests. Physiotherapist Linda Ulenius participates in the walking tests. Päivi Piirilä recites the clinical lung physiology measurements. Neurofilament analyzes are carried out in Sweden by Henrik Zetterberg and Kaj Blennow, researchers at the University of Gothenburg. ApoE analyzes are performed in Dos Liisa Myllykanka 's laboratory at HUS. Prof. Kai Kaila and graduate student LL Samu Kurki also work as partners. Prof Riitta Lassila, dos Lotta JoutsiKorhonen and Tuukka Helin from HUSLAB and LT Mirka Sivula from ATeK's intensive care unit are responsible for analyzing the coagulation studies.

**VI Ethical considerations**

The project’s primary goal is to support patients recovering from critical illnesses. This has been the goal of the outpatient clinic for years, but right now, when the activity is most needed, logistical reasons can’t organize it other than as a research project. Therefore, participation in the project is only possible if the patient gives informed consent. These are often working-age patients whose return to everyday life may be difficult due to the illness. Getting sick with COVID-19 and its consequences is, in addition to its somatic challenges, psychologically burdensome and even stigmatizing. Experiences during intensive care can, at worst, lead to a post-traumatic stress reaction, impaired functioning, and a deteriorated quality of life. The project aims to identify disadvantages and allow patients to discuss matters related to their intensive care and current symptoms. If necessary, they will be referred to rehabilitation and treatment based on the findings. In particular, neurological and neuropsychological signs, as well as cardiac symptoms, are vital for recovery. This is very important in my opinion. All collected information is treated confidentially and analyzed in a pseudonymized manner without the patients being able to be identified at any stage. Clinically significant findings appearing in brain magnetic resonance imaging or other examinations are brought to the attention of the person in charge of the study, who organizes an appropriate assessment and, if necessary, referral to treatment. Regarding the results of heart imaging, a cardiologist is consulted if necessary, and further examinations requiring radiation (CT of the coronary arteries) are performed based on the cardiologist's assessment. An ApoE analysis can reveal an increased risk of memory disease, which can be difficult for the subject to handle. For this reason, the research subject who consents to this sub-study will be given separate information about the study and has the right to choose whether he wants to be informed about his research results. This is asked in the consent form, which also states the subject's right to change his mind about the matter later. Subjects receiving information about their increased risk are allowed to discuss the case with a neurologist.

In my opinion, what is ethically challenging is that the interpretation of the results of neuropsychological testing is highly dependent on language skills. Therefore, only patients whose working language is Finnish can be included in the neuropsychological part of the study. However, the proportion of patients with other languages in the COVID-19 intensive care unit has been considerable. If the project finds that there are significant findings among Finnish-speaking patients, the aim is to organize the study, at least partially, also for willing patients of other languages on clinical grounds. Another ethical challenge is that the logistically possible examination time is only six months after becoming ill, when the patient may have had symptoms for a long time in the worst case. However, the situation with the COVID-19 disease in the summer of 2020 is difficult to predict, and, likely, access to the hospital's facilities and staff resources will be limited, for example, three months after the intensive care of the first patients. Fortunately, the patient can tell about their bothersome symptoms as early as three months in connection with the call and thus get help earlier.

**VII Importance of research**

In the project, the intensive care patient is met after intensive care in the recovery phase, and his situation is examined holistically and individually. We try to answer the patient's questions and refer him to treatment or rehabilitation if necessary. Surviving the intensive care unit alive is not enough; the patient must also be supported in recovery. The particular problems after intensive care are often unfamiliar to the healthcare professionals the patient encounters after discharge from the hospital—the post-hospital clinic functions at its best as a bridge between specialized medical care and primary health care. In addition, the study provides valuable new information about the patient’s cardiovascular health after contracting the coronavirus infection. The information obtained in heart examinations and coagulation studies may lead to medication changes, which aim to prevent complications. Information about neurological morbidity obtained during long-term follow-up can speed up patients' access to treatment and rehabilitation promptly.

The project involves extensive cooperation between medical specialties and different disciplines. We will also utilize and strengthen the established collaboration between neuropsychology and intensive care researchers. The project also includes international cooperation and supports the progress of academic career development through the departments of three doctoral projects (two in intensive care medicine and one in neuropsychology). In collaboration with the University of Helsinki, within the framework of the project, the advanced studies of 1-2 Bachelor of Medicine students and the pro-grad work of 1-2 neuropsychology students will be supervised.

References :

1) Pandharipande P, Girard T, Jackson J, et al.: Long-term Cognitive impairment after critical

2) Calsavara A, Costa P, Nobre V, et al.: Factors associated with short and long-term Cognitive changes in patients with sepsis. Scientific Reports 2018 8:4509

3) Herridge MS, Cheung AM, Tansey CM, et al.: One-year outcomes in acute respiratory distress syndrome survivors. NEJM 2003; 348:683-93

4) Herridge MS, Tansey CM, Matté A, et al.: Functional disability five years after acute respiratory distress syndrome. N Engl J Med. 2011 Apr 7;364:1293 -304. doi : 10.1056/NEJMoa101180

5) Mikkelsen ME, Christie JD, Lanken PN, et al.: The adult respiratory distress syndrome Cognitive outcomes study: long-term neuropsychological function in survivors of acute lung injury. Am J Respir Crit Care Med. 2012 Jun 15;185(12):1307-15. DOI : 10.1164/rccm.201111-2025OC.

6) Asadi-Pooya AA1, Simani L: Central nervous system manifestations of COVID-19: A systematic review. J Neurol Sci. 2020 Apr 11;413:116832 . doi : 10.1016/j.jns.2020.116832.

7) Mao L, Jin H, Wang M, et al.: Neurologic manifestations of hospitalized patients with coronavirus disease 2019 in Wuhan, China. JAMA Neurol 2020 doi : 10.1001/jamaneurol.2020.1127

8) Carod -Artal FJ: Neurological complications of coronavirus and COVID-19. Rev Neurol. 2020 May 1;70(9):311-322. doi : 10.33588/rn.7009.2020179.

9) Helms J, Kremer S, Merdji H, et al.: Neurologic features in severe SARS-CoV2 infection. NEJM 2020, DOI: 10.1056/NEJMc2008597.

10) Wihersaari L, Ashton NJ, Reinikainen M, Jakkula P, Pettilä V, Hästbacka J, et al.: Neurofilament light as an outcome predictor after cardiac arrest: a post hoc analysis of the COMACARE trial. Intensive Care Med 2020: https://doi.org/10.1007/s00134-020-06218-9

11) Khalil M, Teunissen CE, Otto M, Piehl F, Sormani MP, Gattringer T, et al. : Neurofilaments as biomarkers in neurological disorders Nat Rev Neurol. 2018 Oct;14(10):577-589.

12) Kanberg N, Ashton NJ, Andersson LM, Yilmaz A, Lindh M, Nilsson S, et al.: Neurochemical evidence of astrocytic and neuronal injury commonly found in COVID-19. Neurology. 2020 Jun 16:10.1212/WNL.0000000000010111. DOI : 10.1212/WNL.0000000000010111.

13) Kuo CL, Pilling LC, Atkins JL, Kuchel GA, Melzer D. ApoE e2 and aging-related outcomes in 379,000 UK Biobank participants. medRxiv [Internet]. 2020. http://medrxiv.org/content/early/2020/02/13/2020.02.12. 20022459.abstract. Accessed May 4, 2020

14) Kuo CL, Pilling L, Atkins JL , Masoli JAH, Delgado J, Kuchel GA, et al.: APOE e4 Genotype Predicts Severe COVID-19 in the UK Biobank Community Cohort. J Gerontol A Biol Sci Med Sci, 2020, Vol. XX, No. XX, 1–2 doi:10.1093/ Gerona / glaa131

15) Vrsalovic M, Vrsalovic Presecki A. Cardiac troponins predict mortality in patients with COVID-19: A meta-analysis of adjusted risk estimates. J Infect. 2020;S 0163-4453(20)30300-5.

16) Petrilli CM, Jones SA, Yang J, et al. Factors associated with hospital admission and critical illness among 5279 people with coronavirus disease 2019 in New York City: prospective cohort study. BMJ. 2020;369:m1966.

17) Siripanthong B, Nazarian S, Muser D, Deo R, Santangeli P, Khanji MY, Cooper LT Jr, Chahal CAA. Recognizing COVID-19-related myocarditis: the possible pathophysiology and proposed guideline for diagnosis and management. Heart Rhythm. 2020 May 5.

18) Szekely Y, Lichter Y, Taieb P, et al. The Spectrum of Cardiac Manifestations in Coronavirus Disease 2019 (COVID-19) - a Systematic Echocardiographic Study [published online ahead of print, 2020 May 29]. Circulation. 2020

19) Tang N, Bai H, Chen X, et al . Anticoagulant treatment is associated with decreased mortality in severe coronavirus disease 2019 patients with coagulopathy. J Thromb Haemost 2020;18:1094 ¤#x2013;9.

20) Zhang L, Yan X, Fan Q, et al . D-dimer levels on admission to predict in-hospital mortality in patients with Covid-19. J Thromb Haemost , published online 19 April 2020. DOI:10.1111/jth.14859.

21) Whitcroft KL, Hummel T. Olfactory Dysfunction in COVID-19. Diagnosis and Management. REST. Published online May 20, 2020. doi:10.1001/jama.2020.8391

22) Philpott C, DeVere D. 2014. Postinfectious and post-traumatic olfactory disorders. In: Welge-Luessen A, Hummel T, editors. Management of smell and taste disorders. New York: Thieme ; pp. 91–105.

23) Sorokowska A, Drechsler E, Karwowski M, Hummel T. Effects of olfactory training: a meta-analysis. Rhinology 2017 Mar 1;55(1):17-26.

24) Rombaux P, Huart C, Deggouj N, Duprez T, Hummel T. Prognostic Value of Olfactory Bulb Volume Measurement for Recovery in Postinfectious and Posttraumatic Olfactory Loss. Otolaryngol Head Neck Surg 2012 Dec;147(6):1136-41.

25) Ueland T, Holter JC, Holten AR, Müller KE, Lind A, Bekken GK, et al.: Distinct and early increase in circulating MMP-9 in COVID-19 patients with respiratory failure. J Infect 2020; 81:e 41-e43

26) Lauhio A, Konttinen YT, Tschesche H, Nordström D, Salo T, Lähdevirta J, et al.: Reduction of matrix metalloproteinase 8-neutrophil collagenase levels during long-term doxycycline treatment of reactive arthritis. Antimicrob Agents Chemother 1994; 38: 400-2

27) Sivula M, Hästbacka J, Kuitunen A, Lassila R, Tervahartiala T, Sorsa T, et al.: Systemic matrix metalloproteinase-8 and tissue inhibitor of metalloproteinases-1 levels in severe sepsis-associated coagulopathy. Acta Anaesthesiol Scand 2015;59: 176-84

28) Hästbacka J, Linko R, Tervahartiala T, Varpula T, Hovilehto S, Parviainen I, et al.: Serum MMP-8 and TIMP-1 in critically ill patients with acute respiratory failure: TIMP-1 is associated with increased 90- day mortality. Anesth Analg . 2014;118:790 -8.

29) Schuster A, Hor KN, Kowallick JT, Beerbaum P, Kutty S. Cardiovascular Magnetic Resonance Myocardial Feature Tracking: Concepts and Clinical Applications. Circ Cardiovasc Imaging. 2016; 9th 004077.

30) Patel AR, Kramer CM. Role of Cardiac Magnetic Resonance in the Diagnosis and Prognosis of Nonischemic Cardiomyopathy. JACC Cardiovasc Imaging. 2017;10:1180 -1193.

31) Toubiana , J. et al. Outbreak of Kawasaki disease in children during the COVID-19 pandemic: a prospective observational study in Paris, France. Preprint at medRxiv https://doi.org/10.1101/2020.05.10.20097394 (2020)

32) Amanat F, Stadlbauer D, Strohmeier S, Nguyen T, Chromikova V, McMahon M, et al.: A serological assay to detect SARS-CoV-2 seroconversion in humans. Nat Med 2020; 26: 1033-36

33) Haveri A, Smura T, Kuivanen S, Österliund P, Hepojoki J, Ikonen N, et al.: Serological and Molecular findings during SARS-CoV-2 infection: the first case study in Finland, January to February 2020. Euro Survey 2020; 25(11):2000266. doi : 10.2807/1560- 7917.ES.2020.25.11.2000266
